# Efficacy and safety of treatment regimens for patients with metastatic, locally advanced, or recurrent breast cancer carrying *BRCA1/BRCA2* pathogenic variants: A network meta-analysis

**DOI:** 10.3389/fonc.2023.1080297

**Published:** 2023-02-14

**Authors:** Yingxuan Zhu, Yang Li, Weida Liu, Ruozhu Zhou, Lap Ah Tse, Yang Wang, Wei Li

**Affiliations:** ^1^ Medical Research and Biometrics Center, National Center for Cardiovascular Diseases, Fuwai Hospital, Peking Union Medical College & Chinese Academy of Medical Sciences, Beijing, China; ^2^ Peking Union Medical College Hospital, Chinese Academy of Medical Sciences & Peking Union Medical College, Beijing, China; ^3^ Department of Oncology, China-Japan Friendship Hospital, Beijing, China; ^4^ Jockey Club School of Public Health and Primary Care, Faculty of Medicine, The Chinese University of Hong Kong, Hong Kong, Hong Kong SAR, China

**Keywords:** breast neoplasms, genes, BRCA1, BRCA2, network meta-analysis

## Abstract

**Objective:**

Patients with breast cancer carrying *BRCA1* and *BRCA2* genetic alterations show poor prognoses. However, the efficacy of pharmacotherapies for patients with advanced breast cancer carrying *BRCA1/2* pathogenic variants remains unclear. This study aimed to conduct a network meta-analysis to assess the efficacy and safety of various pharmacotherapies for patients with metastatic, locally advanced, or recurrent breast cancer carrying *BRCA1/BRCA2* pathogenic variants.

**Methods:**

A literature search was conducted using Embase, PubMed, and Cochrane Library (CENTRAL), from inception to 11^th^ May 2022. The references of included articles were screened to identify relevant literature. This network meta-analysis included patients with metastatic locally advanced or recurrent breast cancer who received pharmacotherapy and carried deleterious variants of *BRCA1/2*. The Preferred Reporting Items for Systematic Reviews and Meta-analysis (PRISMA) guidelines were followed for conducting and reporting this systematic meta-analysis. The Grading of Recommendations Assessment, Development, and Evaluation (GRADE) method was employed to evaluate evidential certainty. Frequentist random-effect model was applied. Results of objective response rate (ORR), progression-free survival (PFS), overall survival (OS), and rates of any-grade adverse events were presented.

**Results:**

Nine randomized controlled trials were obtained comprising six treatment regimens, including 1912 patients with pathogenic variants of *BRCA1* and *BRCA2.* The orchestration of PARP inhibitors with platinum-based chemotherapy was found to be the most effective with a pooled odds ratio (OR) of 3.52 (95% CI 2.14, 5.78) for ORR; 1.53 (1.34,1.76), 3.05 (1.79, 5.19), and 5.80 (1.42, 23.77) for 3-, 12-, and 24-month PFS, respectively, and 1.04 (1.00, 1.07), 1.76 (1.25, 2.49) and 2.31 (1.41, 3.77) for 3-, 12-, and 36-month OS, respectively compared to those receiving non-platinum-based chemotherapy. However, it posed an elevated risk of some adverse events. Platinum-based chemotherapy alone or PARP inhibitors markedly improved ORR, PFS, and OS compared to non-platinum-based chemotherapy. Interestingly, platinum-based chemotherapy surpassed PARP inhibitors in terms of efficacy. Evidence on programmed death-ligand 1(PD-L1) inhibitors and sacituzumab govitecan (SG) suggested low quality and insignificant results.

**Conclusions:**

Among all treatment regimens, PARP inhibitors with platinum exhibited the best efficacy, although with a trade-off of elevated risk of some types of adverse events. Future research on direct comparisons between different treatment regimens specifically targeting patients with breast cancer carrying *BRCA1/2* pathogenic variants with a pre-specified adequate sample size is warranted.

## Introduction

Breast cancer is the most diagnosed cancer in women and the fifth leading cause of cancer mortality worldwide, with an estimated 685,000 deaths in 2020 (1). Breast cancer is also the leading cause of cancer-related disability-adjusted life years (DALYs) for females globally, as reported in 2019 (2). It has a rapidly rising incidence rate in transitioning countries in South America, Africa, and Asia, as well as high-income Asian countries (1).

Pathogenic variants of breast cancer susceptibility genes 1 or 2 (*BRCA1*/*BRCA2*) reportedly occur in nearly 5% of patients with breast cancer (3, 4). These patients are more likely to have a family history, receive an early diagnosis, or show a worse prognosis, especially at an advanced cancer stage (5, 6). Genetic alterations in *BRCA1*/*BRCA2* cause the weakening of DNA double-strand break (DSB) repair ability, making the tumor cells highly dependent on the pathways involved in single-strand break repair (7, 8). The enzyme, poly(adenosine diphosphate-ribose) polymerase (PARP) crucially controls this pathway, making PARP inhibitors a promising treatment strategy for patients with breast cancer carrying *BRCA1/2* pathogenic variants (9). The U.S. Food and Drug Administration approved two PARP inhibitors, olaparib and talazoparib, as treatment options for patients with metastatic or advanced breast cancer carrying germline *BRCA1/2* pathogenic variants. Other PARP inhibitors have also been tested for breast cancer therapy, including veliparib and niraparib. As a class, PARP inhibitors share some similarities (10). Platinum agents are reportedly more effective for patients with breast cancer carrying germline *BRCA1/2* pathogenic variants (11). These treatments are recommended as preferred treatment options for recurrent or stage IV TNBC in the updated guidelines (12).

Platinum-based chemotherapy and PARP inhibitors are common regimens for patients with breast cancer carrying *BRCA* pathogenic variants. Randomized controlled trials (RCTs) using PARP inhibitors or platinum for treating patients with metastatic, locally advanced, or recurrent breast cancer carrying *BRCA1/2* pathogenic variants have shown efficacy, as evidenced by improved survival duration (13–15). However, the comparative performances of these regimens remain unknown.

A previous network meta-analysis compared the efficacy and safety of various drug regimens for patients with *BRCA*-pathogenic variant-associated breast cancer. However, the primary analysis mixed studies on patients at different disease stages, and no comparative results were provided for patients with advanced breast cancer and *BRCA1/2* pathogenic variants (16).

We undertook this network meta-analysis to assess the efficacy and safety of pharmacotherapies for patients with metastatic, locally advanced, or recurrent breast cancer carrying *BRCA1/2* pathogenic variants.

## Methods

The network meta-analysis was performed following the guidelines of the 2020 Preferred Reporting Items for Systematic Reviews and Meta-Analysis (PRISMA) statement (17).

### Data sources and search strategies

From inception until May 11th, 2022, a systematic literature search was conducted in Embase, PubMed, and Cochrane Library (CENTRAL). To identify relevant studies, we screened the references cited in the included publications. Terms related to breast cancer and its synonyms, *BRCA1/2* pathogenic variants, and RCTs were used (please refer to the detailed search string in Appendix 1).

### Study selection and data extraction

Eligibility criteria included the following: (1) studies of patients with advanced or metastatic breast cancer. (2) Studies targeting patients carrying *BRCA1/2* pathogenic variants or those reporting relevant subgroup results. (3) Studies with chemotherapy or targeted therapies as the treatment strategy. (4) Studies reporting at least one of the following outcomes: objective response rate (ORR), progression-free survival (PFS), or overall survival (OS). (5) Studies with an RCT design.

Exclusion criteria were as follows: (1) studies including patients with *BRCA* methylation. (2) Studies including patients treated with non-platinum-based chemotherapy both in the intervention and control arms. (3) Trials published in languages other than English. Only reports with the most updated results were used to retrieve information for studies derived from the same trial.

The screening was conducted by meticulously reading the titles and abstracts of each potential article, and full texts were scrupulously scrutinized when necessary. The following data were collected: author’s names, publication year, study’s abbreviation, registration number, sample size, *BRCA* pathogenic variant type, the proportion of TNBC patients, patients’ indication, treatment regimens, patients’ median age, and efficacy and safety outcomes. Two investigators (ZY and LY) independently conducted study selection and data extraction. Any disparity was adjudicated by a senior reviewer (LW).

### Outcomes and measures

Efficacy outcomes included ORR and PFS rates at 3-, 12-, and 24 months, and OS rates at 3-, 12-, 24, and 36 months. Raw data on the number of patients experiencing/not experiencing the outcome were obtained from Kaplan-Meier survival curves. The toxicological effects were measured as rates of any-grade adverse events (thrombocytopenia, neutropenia, anemia, leukopenia, nausea, vomiting, diarrhea, constipation, decreased appetite, fatigue, headache, alopecia, and back pain).

### Data analysis and evidential quality assessment

All eligible studies were included in the network meta-analysis utilizing the frequentist method and the random-effects model (18). The network estimates were visualized using net-league tables and forest plots. Odds ratios (ORs) with 95% confidence intervals (CIs) were created to quantify outcomes. The P-score, measuring the degree to which one therapy was guaranteed to be superior compared to its counterparts, was used to rank various treatment regimens (19).

Two independent reviewers (ZY and LY) evaluated the risk of bias in each study using the Cochrane risk of bias tool 2.0 for RCTs. All efficacy outcomes were assessed, and the effect of assignment to intervention was regarded as the effect of interest. The study’s overall risk of bias was divided into three categories as follows: low risk of bias if all domains showed low risk; some concerns if there was at least one domain showing some concerns but not at high risk, and high if there was at least one domain at high risk or multiple domains showing some concerns (20). We applied the Grading of Recommendations Assessment, Development, and Evaluation (GRADE) approach to assess the certainty of the evidence and rated it as high, moderate, low, or very low (21).

Cochran’s Q statistic was decomposed into within-design and between-design values to test the heterogeneity (22). Local inconsistency was tested by splitting and comparing indirect and direct effects, and the former estimates were calculated by back-calculation method (23). We also assessed the transitivity by comparing the distributions of potential effect modifiers across treatment regimens. Comparison-adjusted funnel plots were applied to detect publication biases for direct comparisons with treatment ranked by their P-scores (24). Egger’s regression and Begg’s rank tests were also performed to test for asymmetry in any potential publication biases. To assess the robustness of these results, sensitivity analyses were conducted using the surface under the cumulative ranking curve (SUCRA) values to rank the treatments and excluding studies reporting somatic *BRCA* deleterious variants, as the corresponding patients may not share the same advantage as those carrying germline mutations (12). The R package, netmeta, in R version 4.2.0, was used for data analyses.

## Results

**Figure 1:**
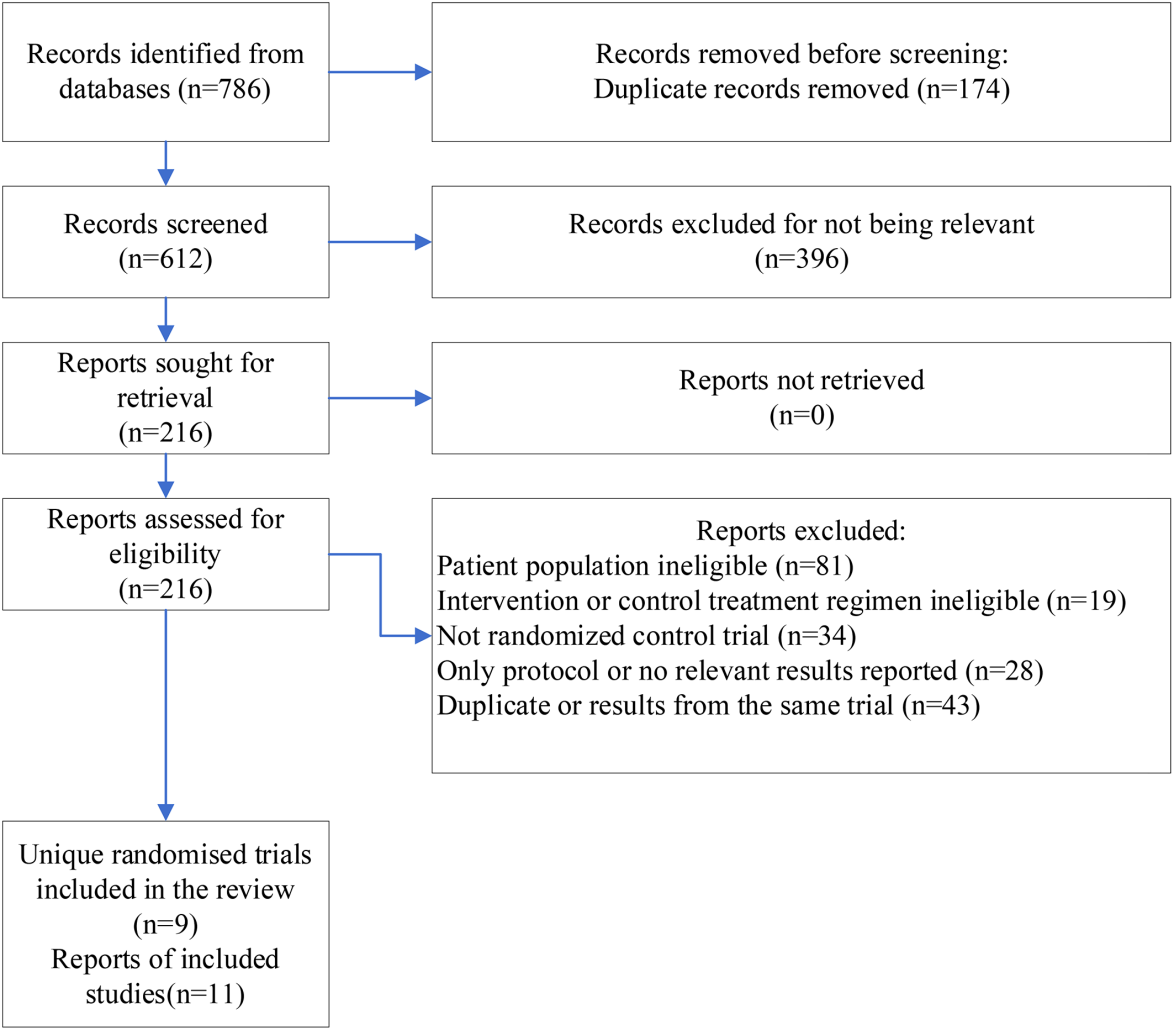
PRISMA flowchart describing the study selection process.

We identified 786 records, and after screening the titles and abstracts, 216 reports were retrieved for screening their full-text (**Figure 1**). Nine RCTs involving 1912 participants with six treatment regimens, including non-platinum-based chemotherapy, platinum-based chemotherapy, PARP inhibitor-containing regimen, PARP inhibitor plus platinum-based chemotherapy, programmed death-ligand 1 (PD-L1) inhibitor, and sacituzumab govitecan (SG), with one multi-arm study, were included. For EMBRACA and OlympiAD studies, additional final analysis reports from updated survival data were included. Finally, 11 reports were included (25–35).

The features of the included studies are summarized in **Table 1**. The range of publications dated from 2018 to 2021, suggesting recent research attention has been drawn toward *BRCA1/2* deleterious variants. Eight studies explicitly reported the results of patients carrying germline *BRCA* deleterious variants, and one reported a mix of patients carrying germline or somatic mutations. Three of the nine studies targeted only TNBC patients. Eight studies provided the outcome as ORR, while another set of eight studies provided the outcome for survival rate as PFS; seven stated the outcome as the OS rate, and five offered comprehensive information on adverse events included in the analysis.

**Table 1:** Characteristics of the included studies.

Study	Study abbreviation	Registration number	Sample size	*BRCA* pathogenic variant type	Proportion of TNBC*	Patients’ indication	Intervention	Control	Outcomes
A. Bardia 2021 (25)	ASCENT	NCT02574455	34	Germline	0.68	metastatic breast cancer	Sacituzumab govitecan	Non-platinum-based chemotherapy	ORR
Nicholas C. Turner 2021 (26)	BRAVO	NCT01905592	206	Germline	0.54	advanced breast cancer	PARP inhibitor	Non-platinum-based chemotherapy	ORR; PFS; OS
Leisha A. Emens 2021 (27)	IMpassion130	NCT02425891	89	Germline or somatic	1	advanced breast cancer	PD-L1 inhibitor	Non-platinum-based chemotherapy	PFS; OS
Véronique Diéras 2020 (28)	BROCADE3	NCT02163694	509	Germline	0.57	metastatic or locally advanced breast cancer	PARP inhibitor + Platinum	Platinum-based chemotherapy	ORR; PFS; OS
J. K. Litton 2020 (29); J. K. Litton 2018 (30)	EMBRACA	NCT01945775	431	Germline	0.44	locally advanced breast cancer or metastatic breast cancer	PARP inhibitor	Non-platinum-based chemotherapy	ORR; PFS; OS
M.E.Robson 2019 (31);Mark Robson 2017 (32)	OlympiAD	NCT02000622	302	Germline	0.5	metastatic breast cancer	PARP inhibitor	Non-platinum-based chemotherapy	ORR; PFS; OS
Andrew Tutt 2018 (33)	TNT	NCT00532727	43	Germline	1	advanced breast cancer	Platinum-based chemotherapy	Non-platinum-based chemotherapy	ORR; PFS; OS
H. S. Han 2018 (34)	BROCADE	NCT01506609	284	Germline	0.41	locally recurrent or metastatic breast cancer	Arm1: PARP inhibitor + Platinum; arm2: PARP inhibitor	Platinum-based chemotherapy	ORR; PFS; OS
J.Zhang 2018 (35)	CBCSG006	NCT01287624	14	Germline	1	metastatic breast cancer	Platinum-based chemotherapy	Non-platinum-based chemotherapy	ORR; PFS

**Figure 2:**
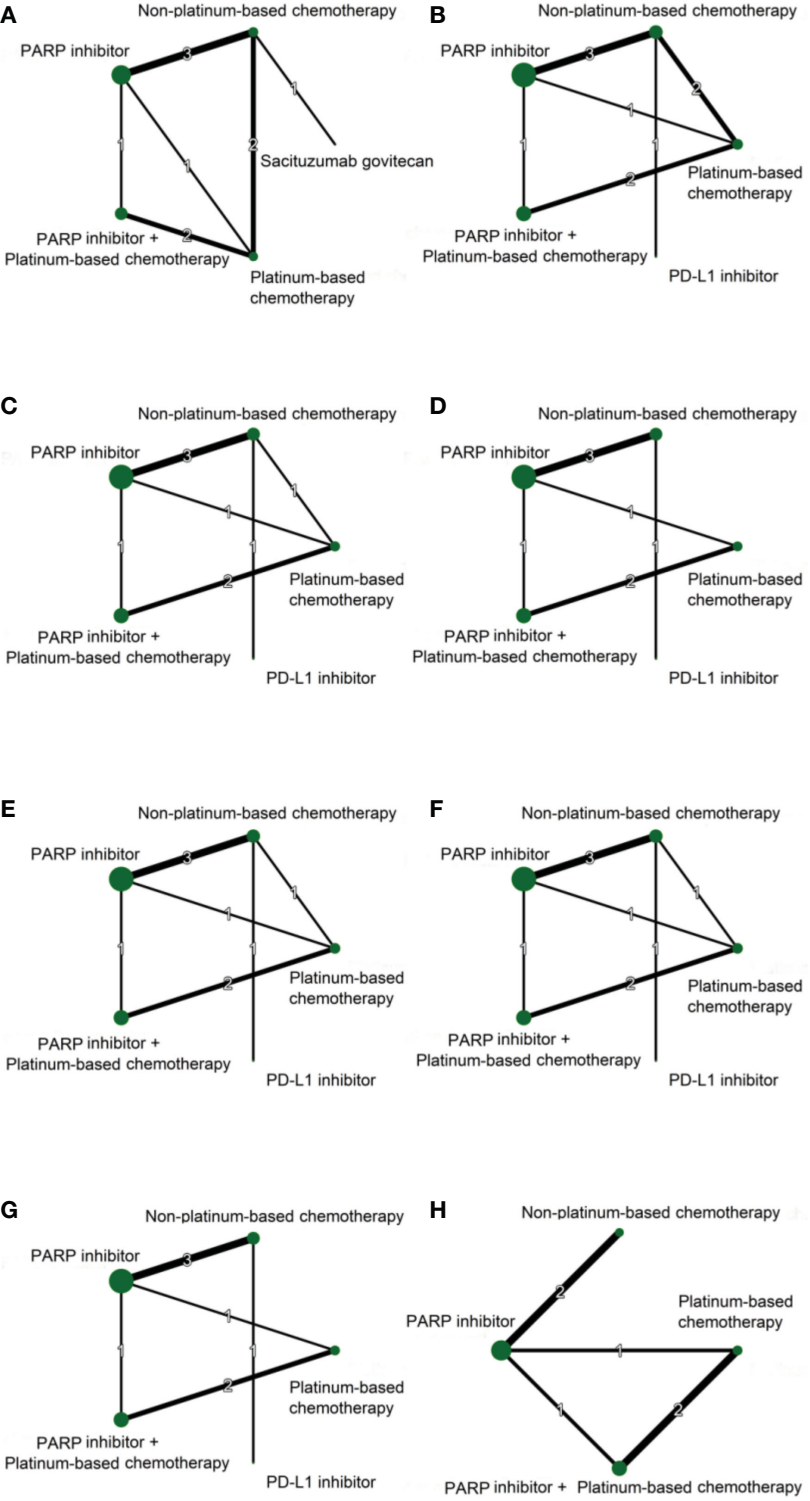
Network plots of direct comparisons for ORR **(A)**, 3-month PFS **(B)**, 12-month PFS **(C)**, 24-month PFS **(D)**, 3-month OS **(E)**, 12-month OS **(F)**, 24-month OS **(G)**, and 36-month OS **(H)** Each node represents a treatment regimen. The thickness of the lines is related to the number of randomized trials that included relevant direct comparisons, and the size of the nodes is proportional to the number of individuals allocated to the corresponding intervention group.

**Figure 2** illustrates the network of available direct comparisons for efficacy outcomes. Network plots for safety outcomes are provided in Appendix 2. **Table 2** shows the network meta-analysis results for the efficacy outcomes of eligible trials. Rankings of efficacy outcomes are shown in **Table 3**.

**Table 2:** League tables of network estimates of odds ratios for efficacy outcome analyses.

(A) objective response rate				
PARP inhibitor + Platinum-based chemotherapy				
1.04 (0.11, 9.93)Ɨ	Sacituzumab govitecan			
1.18 (0.87, 1.60)§	1.13 (0.12, 10.70)Ɨ	Platinum-based chemotherapy		
**2.09 (1.31, 3.34)§**	2.01 (0.22, 18.51)Ɨ	**1.77 (1.15, 2.75)§**	PARP inhibitor	
**3.52 (2.14, 5.78)ǂ**	3.38 (0.37, 30.43)Ɨ	**2.98 (1.89, 4.69)ǂ**	**1.68 (1.24, 2.28)ǂ**	Non-platinum-based chemotherapy

(B) 3-month PFS(lower triangle); 12-month PFS(upper triangle)				
PARP inhibitor + Platinum-based chemotherapy	1.13 (0.88, 1.44)§	**2.02 (1.31, 3.10)§**	2.22 (0.83, 5.94)Ɨ	**3.05 (1.79, 5.19)ǂ**
1.01 (0.98, 1.05)§	Platinum-based chemotherapy	**1.79 (1.16, 2.76)§**	1.97 (0.73, 5.28)Ɨ	**2.70 (1.58, 4.62)ǂ**
**1.20 (1.08, 1.33)§**	**1.18 (1.07, 1.32)§**	PARP inhibitor	1.10 (0.45, 2.68)Ɨ	**1.51 (1.09, 2.08)ǂ**
**1.33 (1.03, 1.71)Ɨ**	**1.32 (1.02, 1.69)Ɨ**	1.11 (0.88, 1.40)Ɨ	PD-L1 inhibitor	1.37 (0.60, 3.14)Ɨ
**1.53 (1.34, 1.76)ǂ**	**1.52 (1.33, 1.74)ǂ**	**1.28 (1.16, 1.41)ǂ**	1.15 (0.93, 1.43)Ɨ	Non-platinum-based chemotherapy

(C) 24-month PFS(lower triangle); 3-month OS(upper triangle)				
PARP inhibitor + Platinum-based chemotherapy	1.05 (0.95, 1.16)Ɨ	1.01 (0.99, 1.02)§	1.00 (0.98, 1.02)§	**1.04 (1.00, 1.07)ǂ**
1.52 (0.19, 12.41)*	PD-L1 inhibitor	0.96 (0.87, 1.06)Ɨ	0.95 (0.86, 1.05)Ɨ	0.99 (0.90, 1.08)Ɨ
1.75 (0.84, 3.63)§	1.15 (0.14, 9.46)Ɨ	Platinum-based chemotherapy	0.99 (0.97, 1.01)§	**1.03 (1.00, 1.06)ǂ**
**3.44 (1.10, 10.72)ǂ**	2.26 (0.39, 13.17)*	1.97 (0.62, 6.27)§	PARP nhibitor	**1.04 (1.01, 1.07)ǂ**
**5.80 (1.42, 23.77)Ɨ**	3.81 (0.81, 18.04)*	3.32 (0.80, 13.84)*	1.69 (0.73, 3.88)ǂ	Non-platinum-based chemotherapy

(D) 12-month OS(lower triangle); 24 month OS(upper triangle)				
PARP inhibitor + Platinum-based chemotherapy	1.06 (0.94, 1.20)§	1.16 (0.66, 2.05)Ɨ	**1.76 (1.25, 2.49)ǂ**	**1.66 (1.24, 2.23)§**
1.06 (0.96, 1.16)§	Platinum-based chemotherapy	1.09 (0.62, 1.94)Ɨ	**1.66 (1.18, 2.36)ǂ**	**1.57 (1.17, 2.11)§**
1.09 (0.81, 1.48)Ɨ	1.03 (0.76, 1.40)Ɨ	PD-L1 inhibitor	1.52 (0.97, 2.39)Ɨ	1.43 (0.88, 2.33)Ɨ
1.16 (0.98, 1.39)ǂ	1.10 (0.93, 1.31)ǂ	1.07 (0.83, 1.37)Ɨ	Non-platinum-based chemotherapy	0.94 (0.79, 1.13)ǂ
**1.22 (1.04, 1.42)§**	1.15 (0.99, 1.35)§	1.12 (0.85, 1.46)Ɨ	1.05 (0.94, 1.16)ǂ	PARP inhibitor

(E) 36-month OS				
PARP inhibitor + Platinum-based chemotherapy
				**1.21 (1.01, 1.46)§**	Platinum-based chemotherapy
**1.77 (1.19, 2.63)§**				1.46 (0.97, 2.19)§	PARP inhibitor
**2.31 (1.41, 3.77)§**	**1.91 (1.16, 3.13)§**			1.31 (0.98, 1.74)§	Non-platinum-based chemotherapy	

The relative effects are measured as OR and 95% CI. All tables list the treatments in the order of p-scores of the treatments for the outcome in the lower triangle. According to GRADE, the certainty of evidence was classified as *very low, Ɨlow, ǂmoderate, and §high. The bold values are the values with statistical significance.

**Table 3:** Network rankings of efficacy outcomes by p-score.

Treatments	ORR	3-month PFS	12-month PFS	24-month PFS	3-month OS	12-month OS	24-month OS	36-month OS
PARP inhibitor + Platinum-based chemotherapy	0.84	0.93	0.94	0.89	0.80	0.89	0.88	0.99
Platinum-based chemotherapy	0.65	0.81	0.77	0.59	0.50	0.63	0.70	0.66
Sacituzumab govitecan	0.66	NA	NA	NA	NA	NA	NA	NA
PARP inhibitor	0.32	0.45	0.40	0.30	0.80	0.11	0.20	0.33
Non-platinum-based chemotherapy	0.03	0.02	0.06	0.05	0.17	0.32	0.07	0.01
PD-L1 inhibitor	NA	0.28	0.33	0.67	0.23	0.54	0.64	NA

P-score values are represented by the numbers. NA indicates no available treatment included for the analysis of the specific outcomes.

### ORR comparison

In terms of ORR, the treatment regimens containing both PARP inhibitors and platinum-based chemotherapies yielded the best benefit versus SG (OR 1.04, 95% CI 0.11 to 9.93), platinum-based chemotherapy (OR 1.18, 95% CI 0.87 to 1.60), PARP inhibitors (OR 2.09, 1.31 to 3.34), and non-platinum-based chemotherapy (OR 3.52, 95% CI 2.14, 5.78). Additionally, platinum-based chemotherapy markedly improved ORR compared to PARP inhibitors (OR 1.77, 95% CI 1.15 to 2.75) and non-platinum-based chemotherapy (OR 1.68, 95% CI 1.24 to 2.28). PARP inhibitors showed a significantly higher ORR compared to non-platinum-based chemotherapy (OR 1.68, 95% CI 1.24 to 2.28).

### PFS comparison

For the outcome of PFS, the treatment regimens containing both PARP inhibitor and platinum-based chemotherapy were most likely to be ranked the best among all treatments. The PFS improved significantly at months 3 (OR 1.20, 95% CI 1.08, 1.33), 12 (OR 2.02, 95% CI 1.31, 3.10), and 24 (OR 3.44, 95% CI 1.10, 10.72) with PARP inhibitor plus platinum compared to other regimens comprising PARP inhibitors alone. The orchestration of PARP inhibitors with platinum-based chemotherapy also showed a significantly better PFS than those of the non-platinum-based chemotherapy at months 3 (OR 1.53, 95% CI 1.34,1.76), 12 (OR 3.05, 95% CI 1.79, 5.19), and 24 (OR 5.80, 95% CI 1.42, 23.77). A significant advantage of PARP inhibitor plus platinum over PD-L1 inhibitor was found for 3-month PFS (OR 1.33 95% CI 1.03, 1.71) but not 12-month (OR 2.22, 95% CI 0.83, 5.94) or 24-month (OR 1.52 95% CI 0.19, 12.41) PFS rates. Furthermore, platinum-based chemotherapy showed significantly higher 3-month and 12-month PFS rates than PARP inhibitor alone (OR 1.18, 95% CI 1.07, 1.32; OR 1.79, 95% CI 1.16, 2.76); however, the relative effect was statistically insignificant for 24-month PFS with a wider confidence interval (OR 1.97, 95% CI 0.62, 6.27). Platinum-based chemotherapy also had a higher 3-month PFS than PD-L1 inhibitor (OR 1.32, 95% CI1.02, 1.69), as well as significantly higher 3-month and 12-month PFS rates than non-platinum-based chemotherapy (OR 1.52, 95% CI 1.33, 1.74; OR 2.70, 95% CI 1.58, 4.62). The treatment regimen containing PARP inhibitors alone showed better 3-month PFS (OR 1.28, 95% CI 1.16, 1.41) and 12-month PFS (OR 1.51, 95% CI 1.09, 2.08) rates than non-platinum-based chemotherapy.

### OS comparison

In terms of the 3-month OS, treatment regimens containing both PARP inhibitors and platinum-based chemotherapy (OR 1.04, 95% CI 1.00, 1.07), platinum-based chemotherapy (OR 1.03, 95% CI 1.00, 1.06), and the treatment using PARP inhibitors (OR 1.04, 95% CI 1.01, 1.07) were significantly superior to non-platinum-based chemotherapy. For the 12-month OS, the treatment regimens containing both PARP inhibitors and platinum demonstrated a significant advantage over treatment with PARP inhibitors alone (OR 1.22, 95% CI 1.04, 1.42). At month 24, the treatment regimen containing both PARP inhibitors and platinum showed a higher OS rate than treatment with PARP inhibitors alone (OR 1.66, 95% CI 1.24, 2.23) and treatment using non-platinum-based chemotherapy (OR 1.76, 1.25, 2.49). Platinum-based chemotherapy also showed a better 24-month OS compared to PARP inhibitors alone (OR 1.57, 95% CI 1.17, 2.11) and non-platinum-based chemotherapy (OR 1.66, 95% CI 1.18, 2.36). For 36-month OS, only four treatments were included in the analysis. The treatment regimen containing both PARP inhibitors and platinum showed a significantly higher 36-month OS rate versus platinum-based chemotherapy (OR 1.21, 95% CI 1.01, 1.46), PARP inhibitors (OR 1.77, 95% CI 1.19, 2.63), and non-platinum-based chemotherapy (OR 2.31, 95% CI 1.41, 3.77). Platinum-based chemotherapy also showed a better 36-month OS compared to non-platinum-based chemotherapy (OR 1.91, 95% CI 1.16, 3.13).

Results from the IMpassion130 trial, whereby some of the patients carried somatic *BRCA* variants, were excluded from the sensitivity analysis. The principal findings were supported by the results of our sensitivity analysis (Appendix 9).

### Safety analysis

**Figure 3:**
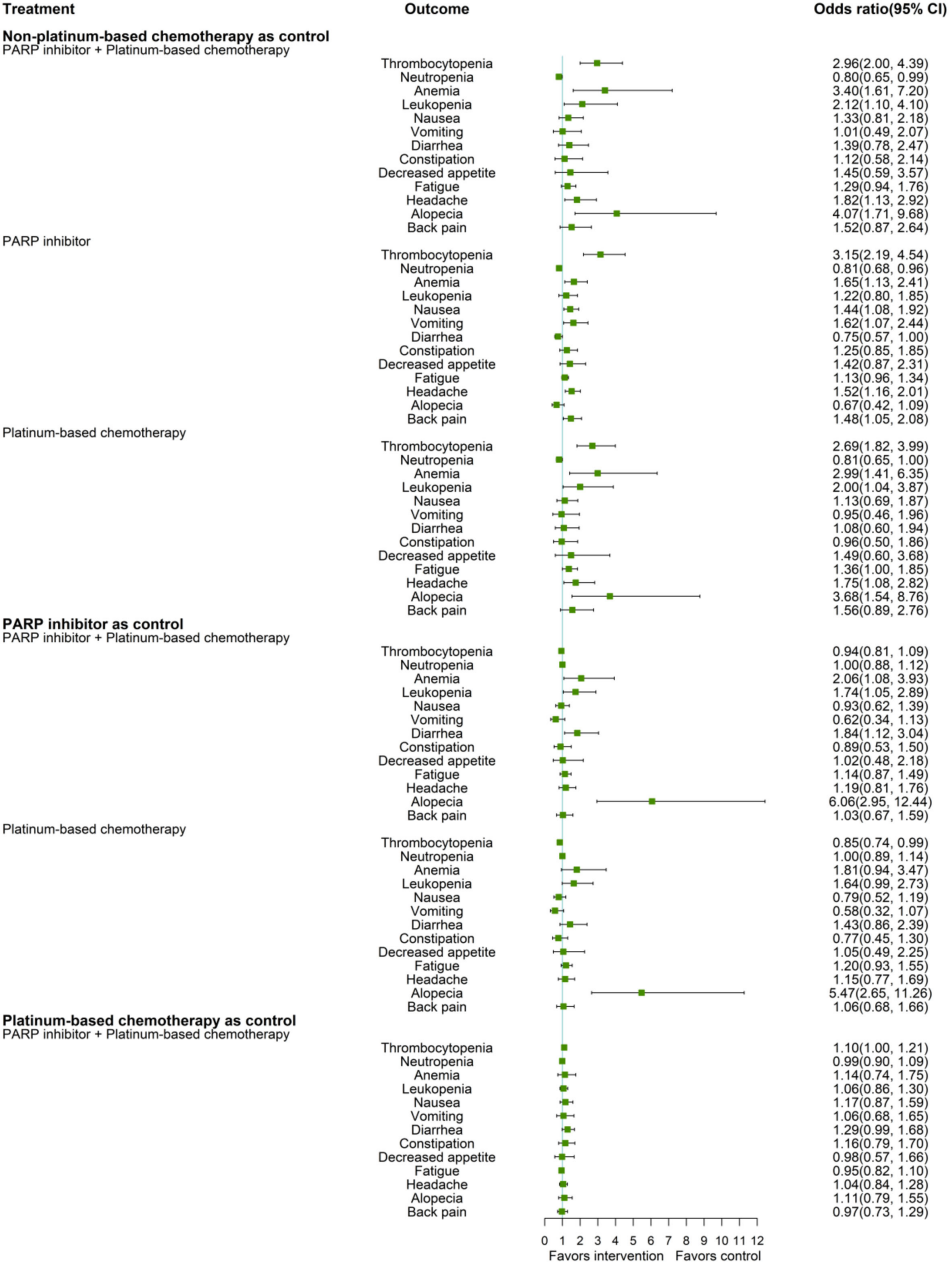
Forest plot for any-grade adverse events.

**Figure 3** shows the network estimates of ORs for adverse events of any grade. Compared to non-platinum-based chemotherapy, the treatment regimen of PARP inhibitor plus platinum had a significantly higher OR for thrombocytopenia (OR 2.96, 95% CI 2.00, 4.39), anemia (OR 3.40, 95% CI 1.61, 7.20), leukopenia (OR 2.12, 95% CI 1.10, 4.10), headache (OR 1.82, 95% CI 1.13, 2.92), and alopecia (OR 4.07, 95% CI 1.71, 9.68), while treatment with PARP inhibitors showed a significantly increased risk of thrombocytopenia (OR 3.15, 95% CI 2.19, 4.54), anemia (OR 1.65, 95% CI 1.13, 2.41), nausea (OR 1.44 95% CI 1.08, 1.92), vomiting (OR 1.62, 95% CI 1.07, 2.44), headache (OR 1.52 95% CI 1.16, 2.01), and back pain (OR 1.48 95% CI 1.05, 2.08), compared to non-platinum-based chemotherapy. Platinum-based chemotherapy had higher ORs for thrombocytopenia (OR 2.69 95% CI 1.82, 3.99), anemia (OR 2.99, 95% CI 1.41, 6.35), leukopenia (OR 2.00, 95% CI 1.04, 3.87), fatigue (OR 1.36, 95% CI 1.00, 1.85), headache (OR 1.75, 95% CI 1.08, 2.82), and alopecia (OR 3.68, 95% CI 1.54, 8.76), compared to non-platinum-based chemotherapy. All three treatment regimens, PARP inhibitor plus platinum (OR 0.8, 95% CI 0.65, 0.99), PARP inhibitor (OR 0.81 95% CI 0.68, 0.96), and platinum-based chemotherapy (OR 0.81 95% CI 0.65, 1.00), had a significantly lower risk of neutropenia than non-platinum-based chemotherapy.

Compared to a treatment regimen containing PARP inhibitor, significantly higher ORs of anemia (OR, 2.06, 95% CI 1.08, 3.93), leukopenia (OR 1.74, 95% CI 1.05, 2.89), diarrhea (OR 1.84, 95% CI 1.12, 3.04), and alopecia (OR 6.06, 95% CI 2.95, 12.44) were observed for the treatment regimens including both PARP inhibitors and platinum.

Platinum-based chemotherapy had a significantly higher OR for alopecia (OR 5.47, 95% CI 2.65, 11.26) than PARP inhibitor but a significantly lower OR for thrombocytopenia (OR 0.85, 95% CI 0.74, 0.99). Compared to platinum-based chemotherapy, the treatment with PARP inhibitors plus platinum showed a substantially higher OR for thrombocytopenia (OR 1.10, 95% CI 1.00, 1.21) but no significant differences were noted for most adverse events.

Supplementary Appendix 3 summarizes the results of the risk of bias assessment. The network meta-analysis’ heterogeneity, intransitivity, inconsistency, and publication bias were also assessed (Supplementary Appendices 4–7). No evidence of significant inconsistency was detected.

## Discussion

This network meta-analysis revealed that incorporating a PARP inhibitor in platinum-based chemotherapy was the most efficient treatment plan for all specified efficacy outcomes, that is ORR, PFS, and OS. Additionally, platinum-based chemotherapy was superior to PARP inhibitor alone in terms of ORR, 3-month PFS, 12-month PFS, and 24-month OS. Among safety outcomes, the treatment regimens comprising both PARP inhibitors and platinum, PARP inhibitor alone, or platinum-based chemotherapy were all associated with a significantly elevated risk for hematological and non-hematological side effects compared to non-platinum-based chemotherapy. The treatment regimen comprising both a PARP inhibitor and platinum showed a higher risk of anemia, leukopenia, diarrhea, and alopecia, compared to PARP inhibitors without platinum; the safety profile to platinum-based chemotherapy was comparable to PARP inhibitor plus platinum. Thus, adding PARP inhibitors to platinum-based chemotherapy would hardly cause more safety burdens.

A previous network meta-analysis of hazard ratios for PFS and ORR found that for patients with advanced breast cancer carrying germline *BRCA* variants, treatment with PARP inhibitor plus platinum were ideal regimens (16). Our study included updated articles and additional treatment regimens. We further evaluated more outcomes of OS and PFS rates at different times and various types of adverse events in detail with a specialized focus on patients with metastatic, locally advanced, or recurrent breast cancer. We found similar results, whereby treatment with PARP inhibitor plus platinum was the most effective. Furthermore, we also identified that platinum-based chemotherapy had a better prognosis in terms of most efficacy outcomes than the treatment with PARP inhibitors alone. Nevertheless, there was only one study that included a direct comparison between platinum-based chemotherapy and PARP inhibitors, which had a major contribution to the pooled results. Further verification in the future is needed.

*BRCA1/2* are crucial for homologous recombination (HR) during DSB repair, and pathogenic variants are linked to genome instability and the progression of cancer (36). It was reported that HR deficiency assays, such as detecting nuclear RAD51 foci in tumor cells, could identify patients with *BRCA* pathogenic variants that are more likely to respond to platinum-containing therapy and PARP inhibitors (37–40). Platinum drugs, like cisplatin and carboplatin, act as DNA cross-linking agents forming intra-strand crosslinks, and in turn inhibiting DNA synthesis, function, and transcription (41). *BRCA* pathogenic variant carriers without sufficient DNA repair ability are, therefore, more sensitive to platinum (42). PARP1 and PARP2 enzymes are critical to the DNA damage response (DDR), and HR deficiency and PARP inhibitors result in synthetic lethality through mechanisms related to catalytic inhibition of the PARP enzyme and trapping of PARP-DNA complexes (9). Our findings showed that the treatment combining both PARP inhibitors and platinum had better efficacy than either regimen alone; however, there may be an increased risk of some adverse events in the former. Since both PARP inhibitors and platinum target and impede DNA synthesis, identifying which of the two treatments is more effective for patients carrying *BRCA* pathogenic variants would be an interesting topic. Although treating patients with advanced breast cancer carrying pathogenic variants of *BRCA* with platinum-based chemotherapy is more advantageous than PARP inhibitors according to our analyses, the results need further verification in a sizable RCT that includes direct comparisons.

Sacituzumab govitecan is a Trop-2-directed antibody-drug conjugate that can increase double-stranded DNA breaks (43). It benefits metastatic TNBC patients regardless of germline *BRCA1/2* variants, as evidenced in an original trial (25). SG was included in the network analysis for ORR comparison, yet the related results were all statistically insignificant, and the evidence was of low certainty. As for PD-L1 inhibitors, the original study also found that *BRCA1/2* status was not a prognostic factor for PFS or OS outcomes (27). Network meta-analysis results for PD-L1 revealed that the quality of evidence was relatively poor. Thus, further investigation is warranted and should be more pertinent to the specific corresponding biomarker (Trop-2 and PD-L1) expression than the *BRCA1/2* variants.

There are also some limitations of our study. First, the sample sizes of several trials were small, which may have led to a broader estimate of the CIs of effects and impaired the evidence quality. This happens mainly because the targeted group with *BRCA1/2* pathogenic variants is only a subgroup from the original trial. Second, not all studies were included for comparing each outcome, as some data were unavailable from the initial studies.

## Conclusion

In conclusion, PARP inhibitor combined with platinum-based chemotherapy was proved as the optimal treatment for patients with metastatic, locally advanced, or recurrent breast cancer carrying *BRCA1/2* pathogenic variants in terms of efficacy outcomes, namely ORR, PFS, and OS. Although the combination of both PARP inhibitor and platinum resulted in more adverse events compared to PARP inhibitor alone and non-platinum-based chemotherapy regimens, which should raise caution in clinical settings, adding PARP inhibitor to platinum barely caused an extra risk of unfavorable events compared to platinum-based chemotherapy alone. Thus, this combined regimen should be considered for patients with advanced breast cancer carrying *BRCA* pathogenic variants for better prognostic outcomes. Confirmatory RCTs of sufficient, pre-specified sample sizes that directly compare currently available treatment regimens and are explicitly aimed at patients carrying *BRCA1/2* pathogenic variants should be conducted in the future.

## Author contributions

WL had full access to all the data in the study and took responsibility for the integrity of the data and the accuracy of the data analysis. YZ: Conceptualization, Methodology, Software, Investigation, Formal analysis, Writing - Original Draft, Visualization. YL: Conceptualization, Investigation, Data Curation Writing - Review & Editing. WDL: Software, Validation, Visualization, Writing - Review & Editing. RZ: Writing - Review & Editing, Conceptualization. LT: Writing - Review & Editing Supervision. YW: Methodology Supervision. WL: Supervision, Writing - Review & Editing, Acquisition of the financial support for the project leading to this publication, Project administration All authors contributed to the article and approved the submitted version.
