# Supplementary material for: Efficacy and safety of treatment regimens for patients with metastatic, locally advanced, or recurrent breast cancer carrying BRCA1/BRCA2 pathogenic variants: A network meta-analysis
Source: Front Oncol. 2023 Feb 14;13:1080297. doi: 10.3389/fonc.2023.1080297 (PMC9971004; doi:10.3389/fonc.2023.1080297)
Supplement: Supplementary file 1 [file DataSheet_1.docx]

**Supplementary appendix**

**Appendix 1 search terms**

("breast cancer" OR "ca breast" OR "breast gland cancer" OR "breast gland neoplasm" OR "breast malignancies" OR "breast malignancy" OR "breast tumor malignant" OR "cancer in the mammary gland" OR "cancer of the breast" OR "cancer of the mammary gland" OR "cancer, breast" OR "malignancies of the breast" OR "malignancy of the breast" OR "malignant breast neoplasm" OR "malignant breast tumor" OR "malignant neoplasm of the breast" OR "malignant tumor of the breast" OR "mamma cancer" OR "mammary cancer" OR "mammary gland cancer" OR "mammary gland malignancy" OR "mammary malignancies" OR "mammary malignancy") AND “BRCA” AND ("randomized controlled trial" OR "controlled trial, randomized" OR "randomised controlled study" OR "randomised controlled trial" OR "randomized controlled study" OR "randomized controlled trial" OR "trial, randomized controlled").

**Appendix 2**: **Network plots of safety outcomes of direct comparisons for thrombocytopenia(A) , neutropenia, anemia, leukopenia, nausea,vomiting, diarrhea, constipation, decreased appetite, fatigue, headache, alopecia, and Back pain(B)**

**
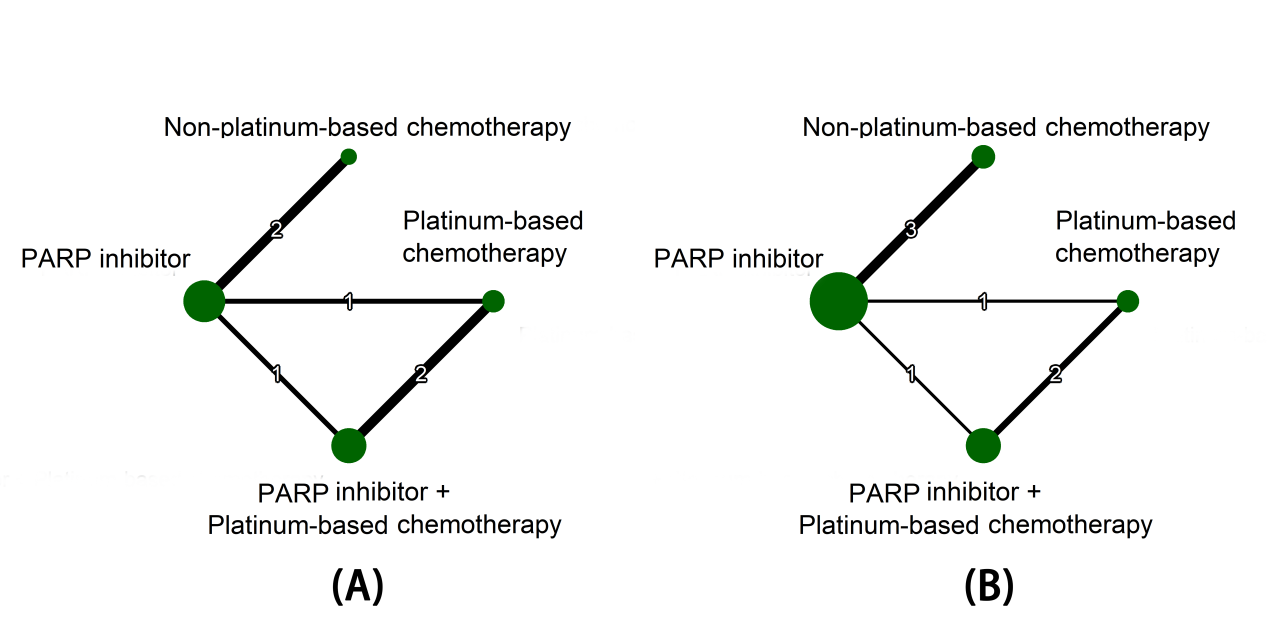
**

Each node represents a treatment regimen. The thickness of the lines is related to the number of randomized trials that evaluated the relevant direct comparison, and the size of the nodes is proportional to the number of individuals allocated to the intervention.

**Appendix 3: Risk of bias assessments using the Revised Cochrane Risk-of-Bias Tool for Randomized Trials(RoB-2)**

| Study | Domain1 | Domain2 | Domain3 | Domain4 | Domain5 | Overall |
| --- | --- | --- | --- | --- | --- | --- |
| ORR | | | | | | |
| Andrew Tutt 2018(TNT) | Low | Low | Low | Some concern | Low | Some concern |
| Véronique Diéras 2020(BROCADE3) | Low | Low | Low | Low | Low | Low |
| Mark Robson 2017( OlympiAD) | Low | Low | Low | Low | Low | Low |
| H. S. Han 2018(BROCADE) | Low | Low | Low | Low | Low | Low |
| J. K. Litton 2018(EMBRACA) | Low | Low | Low | Low | Low | Low |
| J.Zhang 2018(CBCSG006) | Low | Low | Low | Some concern | Some concern | Some concern |
| A. Bardia 2021(ASCENT) | Low | Low | Low | Low | Low | Low |
| Nicholas C. Turner 2021(BRAVO) | Low | Low | Some concern | Low | Low | Some concern |
| PFS-3 month | | | | | | |
| Andrew Tutt 2018(TNT) | Low | Low | Low | Some concern | Low | Some concern |
| Véronique Diéras 2020(BROCADE3) | Low | Low | Low | Low | Low | Low |
| Mark Robson 2017( OlympiAD) | Low | Low | Low | Low | Low | Low |
| H. S. Han 2018(BROCADE) | Low | Low | Low | Low | Low | Low |
| J. K. Litton 2018(EMBRACA) | Low | Low | Low | Low | Low | Low |
| J.Zhang 2018(CBCSG006) | Low | Low | low | Some concern | Some concern | Some concern |
| Nicholas C. Turner 2021(IMpassion130) | Low | Low | Low | Low | Some concern | Some concern |
| Nicholas C. Turner 2021(BRAVO) | Low | Low | Some concern | Low | Low | Some concern |
| PFS-12 month | | | | | | |
| Véronique Diéras 2020(BROCADE3) | Low | Low | Low | Low | Low | Low |
| Mark Robson 2017( OlympiAD) | Low | Low | Low | Low | Low | Low |
| H. S. Han 2018(BROCADE) | Low | Low | Low | Low | Low | Low |
| J. K. Litton 2018(EMBRACA) | Low | Low | Low | Low | Low | Low |
| J.Zhang 2018(CBCSG006) | Low | Low | low | Some concern | Some concern | Some concern |
| Nicholas C. Turner 2021(IMpassion130) | Low | Low | Low | Low | Some concern | Some concern |
| Nicholas C. Turner 2021(BRAVO) | Low | Low | Some concern | Low | Low | Some concern |
| PFS-24 month | | | | | | |
| Véronique Diéras 2020(BROCADE3) | Low | Low | Low | Low | Low | Low |
| Mark Robson 2017( OlympiAD) | Low | Low | Low | Low | Low | Low |
| H. S. Han 2018(BROCADE) | Low | Low | Low | Low | Low | Low |
| J. K. Litton 2018(EMBRACA) | Low | Low | Low | Low | Low | Low |
| Nicholas C. Turner 2021(IMpassion130) | Low | Low | Low | Low | Some concern | Some concern |
| Nicholas C. Turner 2021(BRAVO) | Low | Low | Some concern | Low | Low | Some concern |
| OS-3 month | | | | | | |
| Andrew Tutt 2018(TNT) | Low | Low | Low | Some concern | Low | Some concern |
| Véronique Diéras 2020(BROCADE3) | Low | Low | Low | Low | Low | Low |
| M.E.Robson 2019( OlympiAD) | Low | Low | Low | Low | Low | Low |
| H. S. Han 2018(BROCADE) | Low | Low | Low | Low | Low | Low |
| J. K. Litton 2020(EMBRACA) | Low | Low | Low | Low | Low | Low |
| Nicholas C. Turner 2021(IMpassion130) | Low | Low | Low | Low | Some concern | Some concern |
| Nicholas C. Turner 2021(BRAVO) | Low | Low | Some concern | Low | Low | Some concern |
| OS-12 month | | | | | | |
| Andrew Tutt 2018(TNT) | Low | Low | Low | Some concern | Low | Some concern |
| Véronique Diéras 2020(BROCADE3) | Low | Low | Low | Low | Low | Low |
| M.E.Robson 2019( OlympiAD) | Low | Low | Low | Low | Low | Low |
| H. S. Han 2018(BROCADE) | Low | Low | Low | Low | Low | Low |
| J. K. Litton 2020(EMBRACA) | Low | Low | Low | Low | Low | Low |
| Nicholas C. Turner 2021(IMpassion130) | Low | Low | Low | Low | Some concern | Some concern |
| Nicholas C. Turner 2021(BRAVO) | Low | Low | Some concern | Low | Low | Some concern |
| OS-12 month | | | | | | |
| Andrew Tutt 2018(TNT) | Low | Low | Low | Some concern | Low | Some concern |
| Véronique Diéras 2020(BROCADE3) | Low | Low | Low | Low | Low | Low |
| M.E.Robson 2019( OlympiAD) | Low | Low | Low | Low | Low | Low |
| H. S. Han 2018(BROCADE) | Low | Low | Low | Low | Low | Low |
| J. K. Litton 2020(EMBRACA) | Low | Low | Low | Low | Low | Low |
| Nicholas C. Turner 2021(IMpassion130) | Low | Low | Low | Low | Some concern | Some concern |
| Nicholas C. Turner 2021(BRAVO) | Low | Low | Some concern | Low | Low | Some concern |
| OS-24 month | | | | | | |
| Véronique Diéras 2020(BROCADE3) | Low | Low | Low | Low | Low | Low |
| M.E.Robson 2019( OlympiAD) | Low | Low | Low | Low | Low | Low |
| H. S. Han 2018(BROCADE) | Low | Low | Low | Low | Low | Low |
| J. K. Litton 2020(EMBRACA) | Low | Low | Low | Low | Low | Low |
| Nicholas C. Turner 2021(IMpassion130) | Low | Low | Low | Low | Some concern | Some concern |
| Nicholas C. Turner 2021(BRAVO) | Low | Low | Some concern | Low | Low | Some concern |
| OS-36 month | | | | | | |
| Véronique Diéras 2020(BROCADE3) | Low | Low | Low | Low | Low | Low |
| M.E.Robson 2019( OlympiAD) | Low | Low | Low | Low | Low | Low |
| H. S. Han 2018(BROCADE) | Low | Low | Low | Low | Low | Low |
| J. K. Litton 2020(EMBRACA) | Low | Low | Low | Low | Low | Low |

**Appendix 4: Heterogeneity assessments**

| Outcomes | Total and design-specific within design Q statistics | Q statistic | Degree of freedom | P value |
| --- | --- | --- | --- | --- |
| ORR |  |  |  |  |
|  | Non-platinum-based chemotherapy:PARP inhibitor | 6.026516388 | 2 | **0.049131338** |
|  | Non-platinum-based chemotherapy:Platinum-based chemotherapy | 0.019698941 | 1 | 0.888381157 |
|  | Within designs | 6.046215329 | 3 | 0.10938327 |
| PFS-3 month |  |  |  |  |
|  | Non-platinum-based chemotherapy:PARP inhibitor | 1.142837167 | 2 | 0.564723763 |
|  | Non-platinum-based chemotherapy:Platinum-based chemotherapy | 0.054941371 | 1 | 0.814677752 |
|  | Within designs | 1.197778538 | 3 | 0.753537159 |
| PFS-12 month |  |  |  |  |
|  | Non-platinum-based chemotherapy:PARP inhibitor | 5.790834246 | 2 | 0.055275963 |
|  | Within designs | 5.790834246 | 2 | 0.055275963 |
| PFS-24 months |  |  |  |  |
|  | Non-platinum-based chemotherapy:PARP inhibitor | 8.814006487 | 2 | **0.012191659** |
|  | Within designs | 8.814006487 | 2 | **0.012191659** |
| OS-3 month |  |  |  |  |
|  | Non-platinum-based chemotherapy:PARP inhibitor | 0.139837992 | 2 | 0.932469351 |
|  | Within designs | 0.139837992 | 2 | 0.932469351 |
| OS-12 month |  |  |  |  |
|  | Non-platinum-based chemotherapy:PARP inhibitor | 1.737524045 | 2 | 0.419470523 |
|  | Within designs | 1.737524045 | 2 | 0.419470523 |
| OS-24 month |  |  |  |  |
|  | Within designs | 0.336477167 | 2 | 0.845152171 |
| OS-36 month |  |  |  |  |
|  | Non-platinum-based chemotherapy:PARP inhibitor | 3.80E-06 | 1 | 0.998444291 |
|  | Within designs | 3.80E-06 | 1 | 0.998444291 |
| Thrombocytopenia |  |  |  |  |
|  | Non-platinum-based chemotherapy:PARP inhibitor | 0.4108247 | 1 | 0.521551217 |
|  | PARP inhibitor + Platinum-based chemotherapy:Platinum-based chemotherapy | 6.47E-30 | 0 | NA |
|  | Within designs | 0.4108247 | 1 | 0.521551217 |
| Neutropenia |  |  |  |  |
|  | Non-platinum-based chemotherapy:PARP inhibitor | 11.25989052 | 2 | 0.003588772 |
|  | PARP inhibitor + Platinum-based chemotherapy:Platinum-based chemotherapy | 1.19E-28 | 0 | NA |
|  | Within designs | 11.25989052 | 2 | 0.003588772 |
| Anemia |  |  |  |  |
|  | Non-platinum-based chemotherapy:PARP inhibitor | 18.6523699 | 2 | 8.91E-05 |
|  | Non-platinum-based chemotherapy:PARP inhibitor | 0.052876675 | 1 | 0.818131176 |
|  | Non-platinum-based chemotherapy:PARP inhibitor + Non-platinum-based chemotherapy | 18.82779423 | 1 | 1.43E-05 |
|  | Within designs | 18.6523699 | 2 | 8.91E-05 |
| Leukopenia |  |  |  |  |
|  | Non-Platinum-based chemotherapy:PARP inhibitor | 0.054204048 | 1 | 0.815903024 |
|  | PARP inhibitor + Platinum-based chemotherapy:Platinum-based chemotherapy | 6.76E-32 | 0 | NA |
|  | Within designs | 0.054204048 | 1 | 0.815903024 |
| Fatigue |  |  |  |  |
|  | Non-platinum-based chemotherapy:PARP inhibitor | 0.600219783 | 2 | 0.740736815 |
|  | PARP inhibitor + Platinum-based chemotherapy:Platinum-based chemotherapy | 1.33E-31 | 0 | NA |
|  | Within designs | 0.600219783 | 2 | 0.740736815 |
| Nausea |  |  |  |  |
|  | Non-platinum-based chemotherapy:PARP inhibitor | 11.14155499 | 2 | 0.003807519 |
|  | PARP inhibitor + Platinum-based chemotherapy:Platinum-based chemotherapy | 1.81E-30 | 0 | NA |
|  | Within designs | 11.14155499 | 2 | 0.003807519 |
| Vomiting |  |  |  |  |
|  | Non-platinum-based chemotherapy:PARP inhibitor | 6.577174173 | 2 | 0.037306523 |
|  | PARP inhibitor + Platinum-based chemotherapy:Platinum-based chemotherapy | 2.47E-30 | 0 | NA |
|  | Within designs | 6.577174173 | 2 | 0.037306523 |
| Headache |  |  |  |  |
|  | Non-platinum-based chemotherapy:PARP inhibitor | 1.516573196 | 2 | 0.468468414 |
|  | PARP inhibitor + Platinum-based chemotherapy:Platinum-based chemotherapy | 7.06E-31 | 0 | NA |
|  | Within designs | 1.516573196 | 2 | 0.468468414 |
| Diarrhea |  |  |  |  |
|  | Non-platinum-based chemotherapy:PARP inhibitor | 3.761111495 | 2 | 0.152505328 |
|  | PARP inhibitor + Platinum-based chemotherapy:Platinum-based chemotherapy | 5.92E-31 | 0 | NA |
|  | Between designs | 0.167391866 | 1 | 0.682440217 |
| Constipation |  |  |  |  |
|  | Non-platinum-based chemotherapy:PARP inhibitor | 4.992549627 | 2 | 0.082391351 |
|  | PARP inhibitor + Platinum-based chemotherapy:Platinum-based chemotherapy | 1.76E-31 | 0 | NA |
|  | Within designs | 4.992549627 | 2 | 0.082391351 |
| Decreased appetite |  |  |  |  |
|  | Non-platinum-based chemotherapy:PARP inhibitor | 6.618171999 | 2 | 0.036549565 |
|  | PARP inhibitor + Platinum-based chemotherapy:Platinum-based chemotherapy | 7.43E-32 | 0 | NA |
|  | Within designs | 6.618171999 | 2 | 0.036549565 |
| Back pain |  |  |  |  |
|  | Non-platinum-based chemotherapy:PARP inhibitor | 0.575271445 | 2 | 0.750034763 |
|  | PARP inhibitor + Platinum-based chemotherapy:Platinum-based chemotherapy | 5.62E-31 | 0 | NA |
|  | Within designs | 0.575271445 | 2 | 0.750034763 |
| Alopecia |  |  |  |  |
|  | Non-platinum-based chemotherapy:PARP inhibitor | 6.429762296 | 1 | 0.01122236 |
|  | PARP inhibitor + Platinum-based chemotherapy:Platinum-based chemotherapy | 2.96E-28 | 0 | NA |
|  | Within designs | 6.429762296 | 1 | 0.01122236 |

**Appendix 5: Intransitivity assessments**

Violin plots and box plots of distributions of the potential effect modifiers among different treatment arms


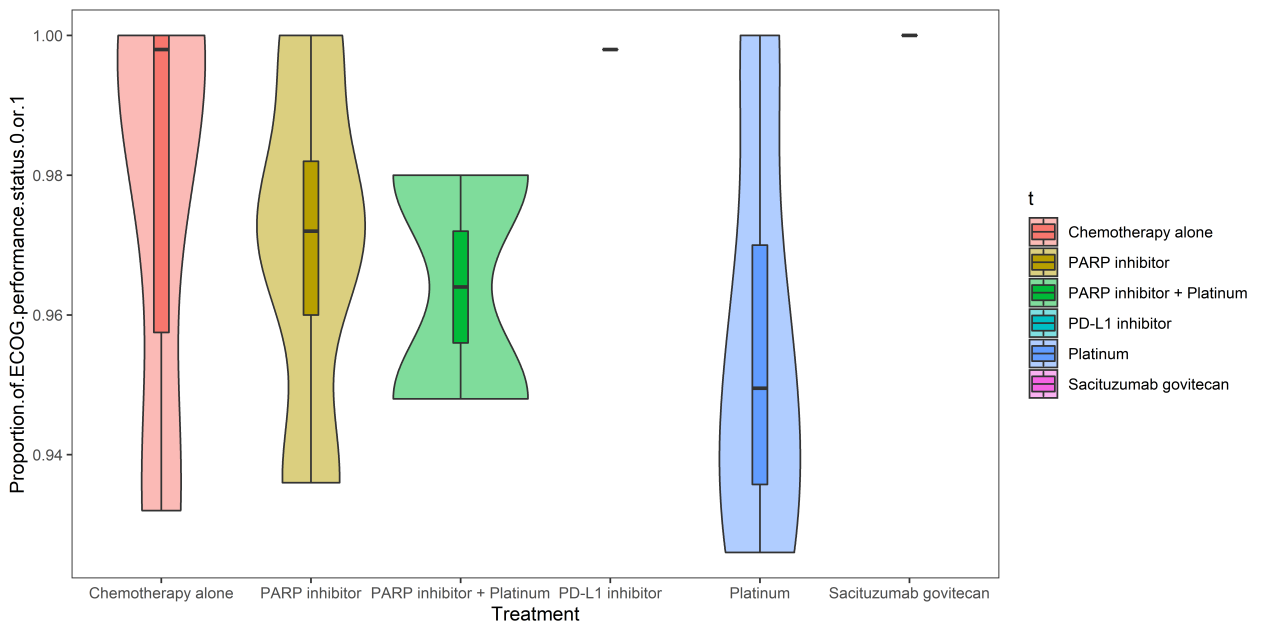


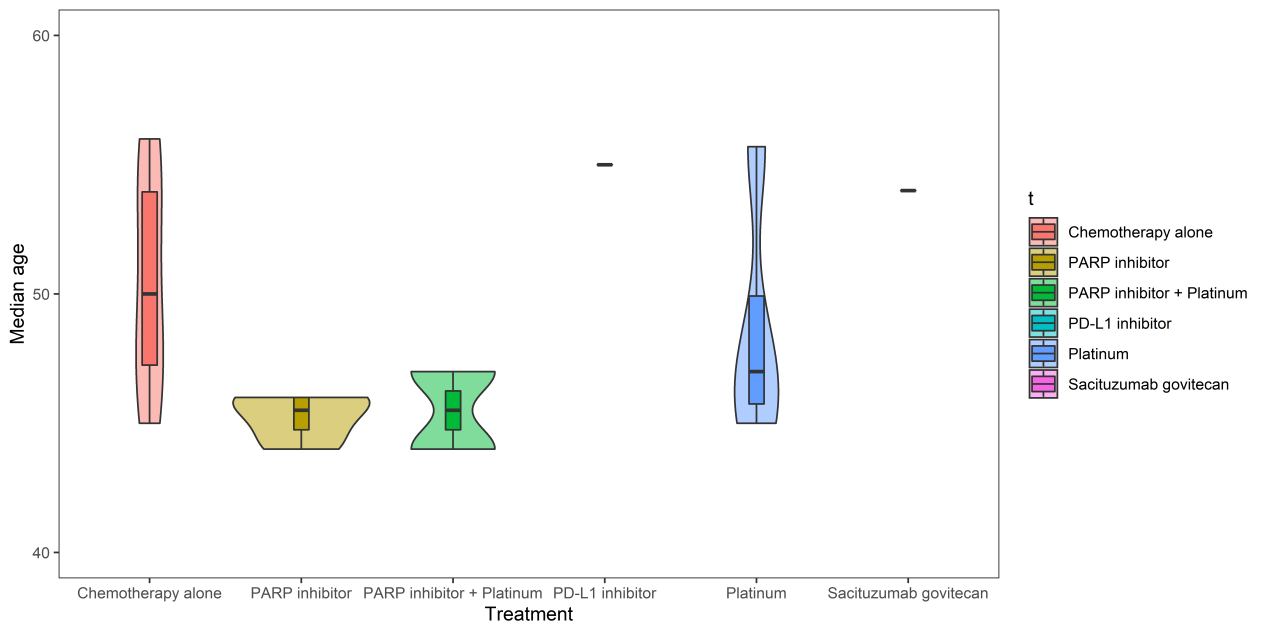


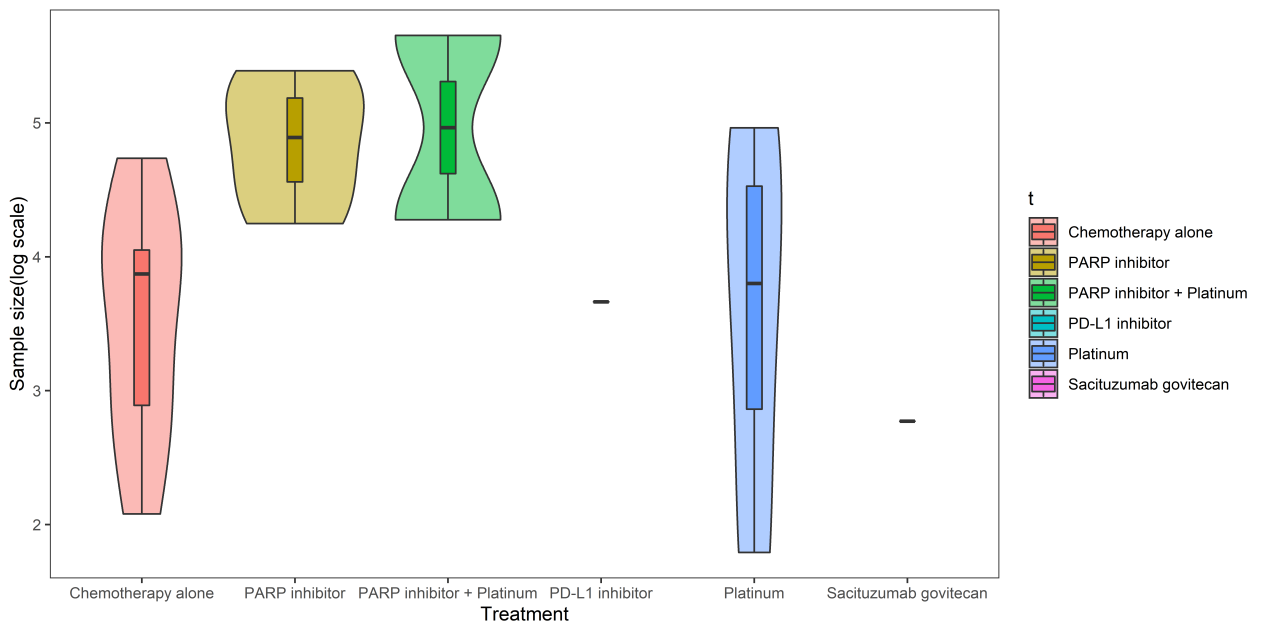


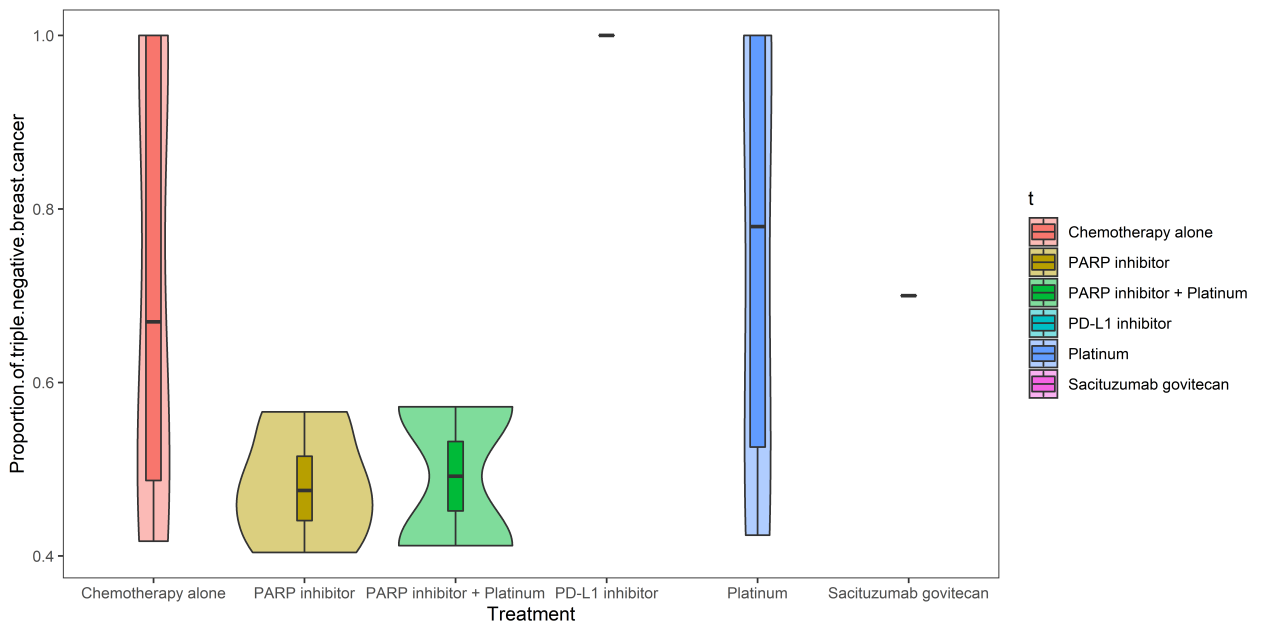


**Appendix 6: Inconsistency(incoherence) assessments**

| Comparisons | Number of studies providing direct evidence | Direct evidence proportion | Network estimates | Direct estimates | Indirect estimates | Inconsistency P-value |
| --- | --- | --- | --- | --- | --- | --- |
| **ORR** |  |  |  |  |  |  |
| PARP inhibitor:Non-Platinum-based chemotherapy-based chemotherapy | 3 | 0.87 | 1.68 ( 1.24 , 2.28 ) | 1.84 ( 1.24 , 2.28 ) | 0.92 ( 1.24 , 2.28 ) | 0.137 |
| PARP inhibitor + Platinum-based chemotherapy-based chemotherapy:Non-Platinum-based chemotherapy-based chemotherapy | 0 | 0 | 3.52 ( 2.14 , 5.78 ) | NA ( 2.14 , 5.78 ) | 3.52 ( 2.14 , 5.78 ) | NA |
| Platinum-based chemotherapy-based chemotherapy:Non-Platinum-based chemotherapy-based chemotherapy | 2 | 0.5 | 2.98 ( 1.89 , 4.69 ) | 2.11 ( 1.89 , 4.69 ) | 4.2 ( 1.89 , 4.69 ) | 0.137 |
| Sacituzumab govitecan:Non-Platinum-based chemotherapy-based chemotherapy | 1 | 1 | 3.38 ( 0.37 , 30.43 ) | 3.38 ( 0.37 , 30.43 ) | NA ( 0.37 , 30.43 ) | NA |
| PARP inhibitor:PARP inhibitor + Platinum-based chemotherapy-based chemotherapy | 1 | 0.69 | 0.48 ( 0.3 , 0.76 ) | 0.37 ( 0.3 , 0.76 ) | 0.85 ( 0.3 , 0.76 ) | 0.104 |
| PARP inhibitor:Platinum-based chemotherapy-based chemotherapy | 1 | 0.57 | 0.56 ( 0.36 , 0.87 ) | 0.47 ( 0.36 , 0.87 ) | 0.73 ( 0.36 , 0.87 ) | 0.327 |
| PARP inhibitor:Sacituzumab govitecan | 0 | 0 | 0.5 ( 0.05 , 4.58 ) | NA ( 0.05 , 4.58 ) | 0.5 ( 0.05 , 4.58 ) | NA |
| PARP inhibitor + Platinum-based chemotherapy-based chemotherapy:Platinum-based chemotherapy-based chemotherapy | 2 | 0.97 | 1.18 ( 0.87 , 1.6 ) | 1.13 ( 0.87 , 1.6 ) | 4.09 ( 0.87 , 1.6 ) | 0.137 |
| PARP inhibitor + Platinum-based chemotherapy-based chemotherapy:Sacituzumab govitecan | 0 | 0 | 1.04 ( 0.11 , 9.93 ) | NA ( 0.11 , 9.93 ) | 1.04 ( 0.11 , 9.93 ) | NA |
| Platinum-based chemotherapy-based chemotherapy:Sacituzumab govitecan | 0 | 0 | 0.88 ( 0.09 , 8.34 ) | NA ( 0.09 , 8.34 ) | 0.88 ( 0.09 , 8.34 ) | NA |
| **PFS-3 month** |  |  |  |  |  |  |
| PARP inhibitor:Non-platinum-based chemotherapy | 3 | 0.92 | 1.28 ( 1.16 , 1.41 ) | 1.28 ( 1.16 , 1.41 ) | 1.24 ( 1.16 , 1.41 ) | 0.857 |
| PARP inhibitor + Platinum-based chemotherapy:Non-platinum-based chemotherapy | 0 | 0 | 1.53 ( 1.34 , 1.76 ) | NA ( 1.34 , 1.76 ) | 1.53 ( 1.34 , 1.76 ) | NA |
| PD-L1 inhibitor:Non-platinum-based chemotherapy | 1 | 1 | 1.15 ( 0.93 , 1.43 ) | 1.15 ( 0.93 , 1.43 ) | NA ( 0.93 , 1.43 ) | NA |
| Platinum-based chemotherapy:Non-platinum-based chemotherapy | 2 | 0.18 | 1.52 ( 1.33 , 1.74 ) | 1.48 ( 1.33 , 1.74 ) | 1.53 ( 1.33 , 1.74 ) | 0.857 |
| PARP inhibitor:PARP inhibitor + Platinum-based chemotherapy | 1 | 0.88 | 0.83 ( 0.75 , 0.93 ) | 0.83 ( 0.75 , 0.93 ) | 0.88 ( 0.75 , 0.93 ) | 0.726 |
| PARP inhibitor:PD-L1 inhibitor | 0 | 0 | 1.11 ( 0.88 , 1.4 ) | NA ( 0.88 , 1.4 ) | 1.11 ( 0.88 , 1.4 ) | NA |
| PARP inhibitor:Platinum-based chemotherapy | 1 | 0.83 | 0.84 ( 0.76 , 0.94 ) | 0.85 ( 0.76 , 0.94 ) | 0.83 ( 0.76 , 0.94 ) | 0.904 |
| PARP inhibitor + Platinum-based chemotherapy:PD-L1 inhibitor | 0 | 0 | 1.33 ( 1.03 , 1.71 ) | NA ( 1.03 , 1.71 ) | 1.33 ( 1.03 , 1.71 ) | NA |
| PARP inhibitor + Platinum-based chemotherapy:Platinum-based chemotherapy | 2 | 1 | 1.01 ( 0.98 , 1.05 ) | 1.01 ( 0.98 , 1.05 ) | 1.07 ( 0.98 , 1.05 ) | 0.857 |
| PD-L1 inhibitor:Platinum-based chemotherapy | 0 | 0 | 0.76 ( 0.59 , 0.98 ) | NA ( 0.59 , 0.98 ) | 0.76 ( 0.59 , 0.98 ) | NA |
| **PFS-12 month** |  |  |  |  |  |  |
| PARP inhibitor:Non-platinum-based chemotherapy | 3 | 0.99 | 1.51 ( 1.09 , 2.08 ) | 1.53 ( 1.09 , 2.08 ) | 0.72 ( 1.09 , 2.08 ) | 0.583 |
| PARP inhibitor + Platinum-based chemotherapy:Non-platinum-based chemotherapy | 0 | 0 | 3.05 ( 1.79 , 5.19 ) | NA ( 1.79 , 5.19 ) | 3.05 ( 1.79 , 5.19 ) | NA |
| PD-L1 inhibitor:Non-platinum-based chemotherapy | 1 | 1 | 1.37 ( 0.6 , 3.14 ) | 1.37 ( 0.6 , 3.14 ) | NA ( 0.6 , 3.14 ) | NA |
| Platinum-based chemotherapy:Non-platinum-based chemotherapy | 1 | 0.04 | 2.7 ( 1.58 , 4.62 ) | 1.32 ( 1.58 , 4.62 ) | 2.79 ( 1.58 , 4.62 ) | 0.583 |
| PARP inhibitor:PARP inhibitor + Platinum-based chemotherapy | 1 | 0.9 | 0.5 ( 0.32 , 0.76 ) | 0.47 ( 0.32 , 0.76 ) | 0.75 ( 0.32 , 0.76 ) | 0.53 |
| PARP inhibitor:PD-L1 inhibitor | 0 | 0 | 1.1 ( 0.45 , 2.68 ) | NA ( 0.45 , 2.68 ) | 1.1 ( 0.45 , 2.68 ) | NA |
| PARP inhibitor:Platinum-based chemotherapy | 1 | 0.87 | 0.56 ( 0.36 , 0.86 ) | 0.57 ( 0.36 , 0.86 ) | 0.51 ( 0.36 , 0.86 ) | 0.873 |
| PARP inhibitor + Platinum-based chemotherapy:PD-L1 inhibitor | 0 | 0 | 2.22 ( 0.83 , 5.94 ) | NA ( 0.83 , 5.94 ) | 2.22 ( 0.83 , 5.94 ) | NA |
| PARP inhibitor + Platinum-based chemotherapy:Platinum-based chemotherapy | 2 | 1 | 1.13 ( 0.88 , 1.44 ) | 1.12 ( 0.88 , 1.44 ) | 4.47 ( 0.88 , 1.44 ) | 0.583 |
| PD-L1 inhibitor:Platinum-based chemotherapy | 0 | 0 | 0.51 ( 0.19 , 1.36 ) | NA ( 0.19 , 1.36 ) | 0.51 ( 0.19 , 1.36 ) | NA |
| **PFS-24 month** |  |  |  |  |  |  |
| PARP inhibitor:Non-platinum-based chemotherapy | 3 | 1 | 1.69 ( 0.73 , 3.88 ) | 1.69 ( 0.73 , 3.88 ) | NA ( 0.73 , 3.88 ) | NA |
| PARP inhibitor + Platinum-based chemotherapy:Non-platinum-based chemotherapy | 0 | 0 | 5.8 ( 1.42 , 23.77 ) | NA ( 1.42 , 23.77 ) | 5.8 ( 1.42 , 23.77 ) | NA |
| PD-L1 inhibitor:Non-platinum-based chemotherapy | 1 | 1 | 3.81 ( 0.81 , 18.04 ) | 3.81 ( 0.81 , 18.04 ) | NA ( 0.81 , 18.04 ) | NA |
| Platinum-based chemotherapy:Non-platinum-based chemotherapy | 0 | 0 | 3.32 ( 0.8 , 13.84 ) | NA ( 0.8 , 13.84 ) | 3.32 ( 0.8 , 13.84 ) | NA |
| PARP inhibitor:PARP inhibitor + Platinum-based chemotherapy | 1 | 0.91 | 0.29 ( 0.09 , 0.91 ) | 0.29 ( 0.09 , 0.91 ) | 0.34 ( 0.09 , 0.91 ) | 0.935 |
| PARP inhibitor:PD-L1 inhibitor | 0 | 0 | 0.44 ( 0.08 , 2.58 ) | NA ( 0.08 , 2.58 ) | 0.44 ( 0.08 , 2.58 ) | NA |
| PARP inhibitor:Platinum-based chemotherapy | 1 | 0.88 | 0.51 ( 0.16 , 1.62 ) | 0.52 ( 0.16 , 1.62 ) | 0.45 ( 0.16 , 1.62 ) | 0.935 |
| PARP inhibitor + Platinum-based chemotherapy:PD-L1 inhibitor | 0 | 0 | 1.52 ( 0.19 , 12.41 ) | NA ( 0.19 , 12.41 ) | 1.52 ( 0.19 , 12.41 ) | NA |
| PARP inhibitor + Platinum-based chemotherapy:Platinum-based chemotherapy | 2 | 1 | 1.75 ( 0.84 , 3.63 ) | 1.75 ( 0.84 , 3.63 ) | NA ( 0.84 , 3.63 ) | NA |
| PD-L1 inhibitor:Platinum-based chemotherapy | 0 | 0 | 1.15 ( 0.14 , 9.46 ) | NA ( 0.14 , 9.46 ) | 1.15 ( 0.14 , 9.46 ) | NA |
| **OS-3 month** |  |  |  |  |  |  |
| PARP inhibitor:Non-platinum-based chemotherapy | 3 | 0.96 | 1.04 ( 1.01 , 1.07 ) | 1.04 ( 1.01 , 1.07 ) | 1.03 ( 1.01 , 1.07 ) | 0.863 |
| PARP inhibitor + Platinum-based chemotherapy:Non-platinum-based chemotherapy | 0 | 0 | 1.04 ( 1 , 1.07 ) | NA ( 1 , 1.07 ) | 1.04 ( 1 , 1.07 ) | NA |
| PD-L1 inhibitor:Non-platinum-based chemotherapy | 1 | 1 | 0.99 ( 0.9 , 1.08 ) | 0.99 ( 0.9 , 1.08 ) | NA ( 0.9 , 1.08 ) | NA |
| Platinum-based chemotherapy:Non-platinum-based chemotherapy | 1 | 0.06 | 1.03 ( 1 , 1.06 ) | 1.02 ( 1 , 1.06 ) | 1.03 ( 1 , 1.06 ) | 0.863 |
| PARP inhibitor:PARP inhibitor + Platinum-based chemotherapy | 1 | 0.87 | 1 ( 0.98 , 1.02 ) | 1 ( 0.98 , 1.02 ) | 1 ( 0.98 , 1.02 ) | 0.888 |
| PARP inhibitor:PD-L1 inhibitor | 0 | 0 | 1.05 ( 0.95 , 1.16 ) | NA ( 0.95 , 1.16 ) | 1.05 ( 0.95 , 1.16 ) | NA |
| PARP inhibitor:Platinum-based chemotherapy | 1 | 0.89 | 1.01 ( 0.99 , 1.03 ) | 1.01 ( 0.99 , 1.03 ) | 1.01 ( 0.99 , 1.03 ) | 0.98 |
| PARP inhibitor + Platinum-based chemotherapy:PD-L1 inhibitor | 0 | 0 | 1.05 ( 0.95 , 1.16 ) | NA ( 0.95 , 1.16 ) | 1.05 ( 0.95 , 1.16 ) | NA |
| PARP inhibitor + Platinum-based chemotherapy:Platinum-based chemotherapy | 2 | 1 | 1.01 ( 0.99 , 1.02 ) | 1.01 ( 0.99 , 1.02 ) | 1.04 ( 0.99 , 1.02 ) | 0.863 |
| PD-L1 inhibitor:Platinum-based chemotherapy | 0 | 0 | 0.96 ( 0.87 , 1.06 ) | NA ( 0.87 , 1.06 ) | 0.96 ( 0.87 , 1.06 ) | NA |
| **OS-12 month** |  |  |  |  |  |  |
| PARP inhibitor:Non-platinum-based chemotherapy | 3 | 0.92 | 0.96 ( 0.86 , 1.06 ) | 0.97 ( 0.86 , 1.06 ) | 0.76 ( 0.86 , 1.06 ) | 0.204 |
| PARP inhibitor + Platinum-based chemotherapy:Non-platinum-based chemotherapy | 0 | 0 | 1.16 ( 0.98 , 1.39 ) | NA ( 0.98 , 1.39 ) | 1.16 ( 0.98 , 1.39 ) | NA |
| PD-L1 inhibitor:Non-platinum-based chemotherapy | 1 | 1 | 1.07 ( 0.83 , 1.37 ) | 1.07 ( 0.83 , 1.37 ) | NA ( 0.83 , 1.37 ) | NA |
| Platinum-based chemotherapy:Non-platinum-based chemotherapy | 1 | 0.28 | 1.1 ( 0.93 , 1.31 ) | 0.92 ( 0.93 , 1.31 ) | 1.18 ( 0.93 , 1.31 ) | 0.204 |
| PARP inhibitor:PARP inhibitor + Platinum-based chemotherapy | 1 | 0.8 | 0.82 ( 0.7 , 0.96 ) | 0.77 ( 0.7 , 0.96 ) | 1.06 ( 0.7 , 0.96 ) | 0.104 |
| PARP inhibitor:PD-L1 inhibitor | 0 | 0 | 0.9 ( 0.68 , 1.18 ) | NA ( 0.68 , 1.18 ) | 0.9 ( 0.68 , 1.18 ) | NA |
| PARP inhibitor:Platinum-based chemotherapy | 1 | 0.7 | 0.87 ( 0.74 , 1.01 ) | 0.85 ( 0.74 , 1.01 ) | 0.9 ( 0.74 , 1.01 ) | 0.761 |
| PARP inhibitor + Platinum-based chemotherapy:PD-L1 inhibitor | 0 | 0 | 1.09 ( 0.81 , 1.48 ) | NA ( 0.81 , 1.48 ) | 1.09 ( 0.81 , 1.48 ) | NA |
| PARP inhibitor + Platinum-based chemotherapy:Platinum-based chemotherapy | 2 | 0.98 | 1.06 ( 0.96 , 1.16 ) | 1.05 ( 0.96 , 1.16 ) | 1.58 ( 0.96 , 1.16 ) | 0.204 |
| PD-L1 inhibitor:Platinum-based chemotherapy | 0 | 0 | 0.97 ( 0.71 , 1.31 ) | NA ( 0.71 , 1.31 ) | 0.97 ( 0.71 , 1.31 ) | NA |
| **OS-24 month** |  |  |  |  |  |  |
| PARP inhibitor:Non-platinum-based chemotherapy | 3 | 1 | 1.06 ( 0.89 , 1.27 ) | 1.06 ( 0.89 , 1.27 ) | NA ( 0.89 , 1.27 ) | NA |
| PARP inhibitor + Platinum-based chemotherapy:Non-platinum-based chemotherapy | 0 | 0 | 1.76 ( 1.25 , 2.49 ) | NA ( 1.25 , 2.49 ) | 1.76 ( 1.25 , 2.49 ) | NA |
| PD-L1 inhibitor:Non-platinum-based chemotherapy | 1 | 1 | 1.52 ( 0.97 , 2.39 ) | 1.52 ( 0.97 , 2.39 ) | NA ( 0.97 , 2.39 ) | NA |
| Platinum-based chemotherapy:Non-platinum-based chemotherapy | 0 | 0 | 1.66 ( 1.18 , 2.36 ) | NA ( 1.18 , 2.36 ) | 1.66 ( 1.18 , 2.36 ) | NA |
| PARP inhibitor:PARP inhibitor + Platinum-based chemotherapy | 1 | 0.93 | 0.6 ( 0.45 , 0.81 ) | 0.58 ( 0.45 , 0.81 ) | 0.94 ( 0.45 , 0.81 ) | 0.413 |
| PARP inhibitor:PD-L1 inhibitor | 0 | 0 | 0.7 ( 0.43 , 1.13 ) | NA ( 0.43 , 1.13 ) | 0.7 ( 0.43 , 1.13 ) | NA |
| PARP inhibitor:Platinum-based chemotherapy | 1 | 0.88 | 0.64 ( 0.47 , 0.86 ) | 0.67 ( 0.47 , 0.86 ) | 0.46 ( 0.47 , 0.86 ) | 0.413 |
| PARP inhibitor + Platinum-based chemotherapy:PD-L1 inhibitor | 0 | 0 | 1.16 ( 0.66 , 2.05 ) | NA ( 0.66 , 2.05 ) | 1.16 ( 0.66 , 2.05 ) | NA |
| PARP inhibitor + Platinum-based chemotherapy:Platinum-based chemotherapy | 2 | 1 | 1.06 ( 0.94 , 1.2 ) | 1.06 ( 0.94 , 1.2 ) | NA ( 0.94 , 1.2 ) | NA |
| PD-L1 inhibitor:Platinum-based chemotherapy | 0 | 0 | 0.91 ( 0.52 , 1.61 ) | NA ( 0.52 , 1.61 ) | 0.91 ( 0.52 , 1.61 ) | NA |
| **OS-36 month** |  |  |  |  |  |  |
| PARP inhibitor:Non-platinum-based chemotherapy | 2 | 1 | 1.31 ( 0.98 , 1.74 ) | 1.31 ( 0.98 , 1.74 ) | NA ( 0.98 , 1.74 ) | NA |
| PARP inhibitor + Platinum-based chemotherapy:Non-platinum-based chemotherapy | 0 | 0 | 2.31 ( 1.41 , 3.77 ) | NA ( 1.41 , 3.77 ) | 2.31 ( 1.41 , 3.77 ) | NA |
| Platinum-based chemotherapy:Non-platinum-based chemotherapy | 0 | 0 | 1.91 ( 1.16 , 3.13 ) | NA ( 1.16 , 3.13 ) | 1.91 ( 1.16 , 3.13 ) | NA |
| PARP inhibitor:PARP inhibitor + Platinum-based chemotherapy | 1 | 0.92 | 0.57 ( 0.38 , 0.84 ) | 0.55 ( 0.38 , 0.84 ) | 0.74 ( 0.38 , 0.84 ) | 0.697 |
| PARP inhibitor:Platinum-based chemotherapy | 1 | 0.85 | 0.69 ( 0.46 , 1.03 ) | 0.71 ( 0.46 , 1.03 ) | 0.57 ( 0.46 , 1.03 ) | 0.697 |
| PARP inhibitor + Platinum-based chemotherapy:Platinum-based chemotherapy | 2 | 1 | 1.21 ( 1.01 , 1.46 ) | 1.21 ( 1.01 , 1.46 ) | NA ( 1.01 , 1.46 ) | NA |
| **Thrombocytopenia** |  |  |  |  |  |  |
| PARP inhibitor:Non-Platinum-based chemotherapy | 2 | 1 | 3.15 ( 2.19 , 4.54 ) | 3.15 ( 2.19 , 4.54 ) | NA ( 2.19 , 4.54 ) | NA |
| PARP inhibitor + Platinum-based chemotherapy:Non-Platinum-based chemotherapy | 0 | 0 | 2.96 ( 2 , 4.39 ) | NA ( 2 , 4.39 ) | 2.96 ( 2 , 4.39 ) | NA |
| Platinum-based chemotherapy:Non-Platinum-based chemotherapy | 0 | 0 | 2.69 ( 1.82 , 3.99 ) | NA ( 1.82 , 3.99 ) | 2.69 ( 1.82 , 3.99 ) | NA |
| PARP inhibitor:PARP inhibitor + Platinum-based chemotherapy | 1 | 0.78 | 1.06 ( 0.92 , 1.23 ) | 1.11 ( 0.92 , 1.23 ) | 0.93 ( 0.92 , 1.23 ) | 0.333 |
| PARP inhibitor:Platinum-based chemotherapy | 1 | 0.77 | 1.17 ( 1.01 , 1.36 ) | 1.12 ( 1.01 , 1.36 ) | 1.34 ( 1.01 , 1.36 ) | 0.333 |
| PARP inhibitor + Platinum-based chemotherapy:Platinum-based chemotherapy | 2 | 1 | 1.1 ( 1 , 1.21 ) | 1.1 ( 1 , 1.21 ) | NA ( 1 , 1.21 ) | NA |
| **Neutropenia** |  |  |  |  |  |  |
| Comparisons | Number of studies providing direct evidence | Direct evidence proportion | Network estimates | Direct estimates | Indirect estimates | Inconsistency P-value |
| PARP inhibitor:Non-Platinum-based chemotherapy | 3 | 1 | 0.81 ( 0.68 , 0.96 ) | 0.81 ( 0.68 , 0.96 ) | NA ( 0.68 , 0.96 ) | NA |
| PARP inhibitor + Platinum-based chemotherapy:Non-Platinum-based chemotherapy | 0 | 0 | 0.8 ( 0.65 , 0.99 ) | NA ( 0.65 , 0.99 ) | 0.8 ( 0.65 , 0.99 ) | NA |
| Platinum-based chemotherapy:Non-Platinum-based chemotherapy | 0 | 0 | 0.81 ( 0.65 , 1 ) | NA ( 0.65 , 1 ) | 0.81 ( 0.65 , 1 ) | NA |
| PARP inhibitor:PARP inhibitor + Platinum-based chemotherapy | 1 | 0.89 | 1 ( 0.89 , 1.14 ) | 1 ( 0.89 , 1.14 ) | 1.04 ( 0.89 , 1.14 ) | 0.826 |
| PARP inhibitor:Platinum-based chemotherapy | 1 | 0.89 | 1 ( 0.88 , 1.12 ) | 1 ( 0.88 , 1.12 ) | 0.96 ( 0.88 , 1.12 ) | 0.826 |
| PARP inhibitor + Platinum-based chemotherapy:Platinum-based chemotherapy | 2 | 1 | 0.99 ( 0.9 , 1.09 ) | 0.99 ( 0.9 , 1.09 ) | NA ( 0.9 , 1.09 ) | NA |
| **Leukopenia** |  |  |  |  |  |  |
| PARP inhibitor:Non-Platinum-based chemotherapy | 2 | 1 | 1.22 ( 0.8 , 1.85 ) | 1.22 ( 0.8 , 1.85 ) | NA ( 0.8 , 1.85 ) | NA |
| PARP inhibitor + Platinum-based chemotherapy:Non-Platinum-based chemotherapy | 0 | 0 | 2.12 ( 1.1 , 4.1 ) | NA ( 1.1 , 4.1 ) | 2.12 ( 1.1 , 4.1 ) | NA |
| Platinum-based chemotherapy:Non-Platinum-based chemotherapy | 0 | 0 | 2 ( 1.04 , 3.87 ) | NA ( 1.04 , 3.87 ) | 2 ( 1.04 , 3.87 ) | NA |
| PARP inhibitor:PARP inhibitor + Platinum-based chemotherapy | 1 | 0.88 | 0.57 ( 0.35 , 0.95 ) | 0.57 ( 0.35 , 0.95 ) | 0.59 ( 0.35 , 0.95 ) | 0.961 |
| PARP inhibitor:Platinum-based chemotherapy | 1 | 0.86 | 0.61 ( 0.37 , 1.01 ) | 0.61 ( 0.37 , 1.01 ) | 0.59 ( 0.37 , 1.01 ) | 0.961 |
| PARP inhibitor + Platinum-based chemotherapy:Platinum-based chemotherapy | 2 | 1 | 1.06 ( 0.86 , 1.3 ) | 1.06 ( 0.86 , 1.3 ) | NA ( 0.86 , 1.3 ) | NA |
| **Anemia** |  |  |  |  |  |  |
| Comparisons | Number of studies providing direct evidence | Direct evidence proportion | Network estimates | Direct estimates | Indirect estimates | Inconsistency P-value |
| PARP inhibitor:Non-Platinum-based chemotherapy | 3 | 1 | 1.65 ( 1.13 , 2.41 ) | 1.65 ( 1.13 , 2.41 ) | NA ( 1.13 , 2.41 ) | NA |
| PARP inhibitor + Platinum-based chemotherapy:Non-Platinum-based chemotherapy | 0 | 0 | 3.4 ( 1.61 , 7.2 ) | NA ( 1.61 , 7.2 ) | 3.4 ( 1.61 , 7.2 ) | NA |
| Platinum-based chemotherapy:Non-Platinum-based chemotherapy | 0 | 0 | 2.99 ( 1.41 , 6.35 ) | NA ( 1.41 , 6.35 ) | 2.99 ( 1.41 , 6.35 ) | NA |
| PARP inhibitor:PARP inhibitor + Platinum-based chemotherapy | 1 | 0.89 | 0.49 ( 0.25 , 0.93 ) | 0.49 ( 0.25 , 0.93 ) | 0.45 ( 0.25 , 0.93 ) | 0.939 |
| PARP inhibitor:Platinum-based chemotherapy | 1 | 0.88 | 0.55 ( 0.29 , 1.06 ) | 0.55 ( 0.29 , 1.06 ) | 0.59 ( 0.29 , 1.06 ) | 0.939 |
| PARP inhibitor + Platinum-based chemotherapy:Platinum-based chemotherapy | 2 | 1 | 1.14 ( 0.74 , 1.75 ) | 1.14 ( 0.74 , 1.75 ) | NA ( 0.74 , 1.75 ) | NA |
| **Nausea** |  |  |  |  |  |  |
| Comparisons | Number of studies providing direct evidence | Direct evidence proportion | Network estimates | Direct estimates | Indirect estimates | Inconsistency P-value |
| PARP inhibitor:Non-Platinum-based chemotherapy | 3 | 1 | 1.44 ( 1.08 , 1.92 ) | 1.44 ( 1.08 , 1.92 ) | NA ( 1.08 , 1.92 ) | NA |
| PARP inhibitor + Platinum-based chemotherapy:Non-Platinum-based chemotherapy | 0 | 0 | 1.33 ( 0.81 , 2.18 ) | NA ( 0.81 , 2.18 ) | 1.33 ( 0.81 , 2.18 ) | NA |
| Platinum-based chemotherapy:Non-Platinum-based chemotherapy | 0 | 0 | 1.13 ( 0.69 , 1.87 ) | NA ( 0.69 , 1.87 ) | 1.13 ( 0.69 , 1.87 ) | NA |
| PARP inhibitor:PARP inhibitor + Platinum-based chemotherapy | 1 | 0.87 | 1.08 ( 0.72 , 1.61 ) | 1.06 ( 0.72 , 1.61 ) | 1.21 ( 0.72 , 1.61 ) | 0.833 |
| PARP inhibitor:Platinum-based chemotherapy | 1 | 0.85 | 1.27 ( 0.84 , 1.91 ) | 1.29 ( 0.84 , 1.91 ) | 1.14 ( 0.84 , 1.91 ) | 0.833 |
| PARP inhibitor + Platinum-based chemotherapy:Platinum-based chemotherapy | 2 | 1 | 1.17 ( 0.87 , 1.59 ) | 1.17 ( 0.87 , 1.59 ) | NA ( 0.87 , 1.59 ) | NA |
| **Vomiting** |  |  |  |  |  |  |
| Comparisons | Number of studies providing direct evidence | Direct evidence proportion | Network estimates | Direct estimates | Indirect estimates | Inconsistency P-value |
| PARP inhibitor:Non-Platinum-based chemotherapy | 3 | 1 | 1.62 ( 1.07 , 2.44 ) | 1.62 ( 1.07 , 2.44 ) | NA ( 1.07 , 2.44 ) | NA |
| PARP inhibitor + Platinum-based chemotherapy:Non-Platinum-based chemotherapy | 0 | 0 | 1.01 ( 0.49 , 2.07 ) | NA ( 0.49 , 2.07 ) | 1.01 ( 0.49 , 2.07 ) | NA |
| Platinum-based chemotherapy:Non-Platinum-based chemotherapy | 0 | 0 | 0.95 ( 0.46 , 1.96 ) | NA ( 0.46 , 1.96 ) | 0.95 ( 0.46 , 1.96 ) | NA |
| PARP inhibitor:PARP inhibitor + Platinum-based chemotherapy | 1 | 0.84 | 1.61 ( 0.89 , 2.91 ) | 1.54 ( 0.89 , 2.91 ) | 2.02 ( 0.89 , 2.91 ) | 0.74 |
| PARP inhibitor:Platinum-based chemotherapy | 1 | 0.82 | 1.71 ( 0.94 , 3.12 ) | 1.8 ( 0.94 , 3.12 ) | 1.38 ( 0.94 , 3.12 ) | 0.74 |
| PARP inhibitor + Platinum-based chemotherapy:Platinum-based chemotherapy | 2 | 1 | 1.06 ( 0.68 , 1.65 ) | 1.06 ( 0.68 , 1.65 ) | NA ( 0.68 , 1.65 ) | NA |
| **Diarrhea** |  |  |  |  |  |  |
| Comparisons | Number of studies providing direct evidence | Direct evidence proportion | Network estimates | Direct estimates | Indirect estimates | Inconsistency P-value |
| PARP inhibitor:Non-Platinum-based chemotherapy | 3 | 1 | 0.75 ( 0.57 , 1 ) | 0.75 ( 0.57 , 1 ) | NA ( 0.57 , 1 ) | NA |
| PARP inhibitor + Platinum-based chemotherapy:Non-Platinum-based chemotherapy | 0 | 0 | 1.39 ( 0.78 , 2.47 ) | NA ( 0.78 , 2.47 ) | 1.39 ( 0.78 , 2.47 ) | NA |
| Platinum-based chemotherapy:Non-Platinum-based chemotherapy | 0 | 0 | 1.08 ( 0.6 , 1.94 ) | NA ( 0.6 , 1.94 ) | 1.08 ( 0.6 , 1.94 ) | NA |
| PARP inhibitor:PARP inhibitor + Platinum-based chemotherapy | 1 | 0.91 | 0.54 ( 0.33 , 0.89 ) | 0.53 ( 0.33 , 0.89 ) | 0.71 ( 0.33 , 0.89 ) | 0.734 |
| PARP inhibitor:Platinum-based chemotherapy | 1 | 0.84 | 0.7 ( 0.42 , 1.17 ) | 0.73 ( 0.42 , 1.17 ) | 0.57 ( 0.42 , 1.17 ) | 0.734 |
| PARP inhibitor + Platinum-based chemotherapy:Platinum-based chemotherapy | 2 | 1 | 1.29 ( 0.99 , 1.68 ) | 1.29 ( 0.99 , 1.68 ) | NA ( 0.99 , 1.68 ) | NA |
| **Constipation** |  |  |  |  |  |  |
| Comparisons | Number of studies providing direct evidence | Direct evidence proportion | Network estimates | Direct estimates | Indirect estimates | Inconsistency P-value |
| PARP inhibitor:Non-Platinum-based chemotherapy | 3 | 1 | 1.25 ( 0.85 , 1.85 ) | 1.25 ( 0.85 , 1.85 ) | NA ( 0.85 , 1.85 ) | NA |
| PARP inhibitor + Platinum-based chemotherapy:Non-Platinum-based chemotherapy | 0 | 0 | 1.12 ( 0.58 , 2.14 ) | NA ( 0.58 , 2.14 ) | 1.12 ( 0.58 , 2.14 ) | NA |
| Platinum-based chemotherapy:Non-Platinum-based chemotherapy | 0 | 0 | 0.96 ( 0.5 , 1.86 ) | NA ( 0.5 , 1.86 ) | 0.96 ( 0.5 , 1.86 ) | NA |
| PARP inhibitor:PARP inhibitor + Platinum-based chemotherapy | 1 | 0.87 | 1.12 ( 0.67 , 1.88 ) | 1.06 ( 0.67 , 1.88 ) | 1.66 ( 0.67 , 1.88 ) | 0.561 |
| PARP inhibitor:Platinum-based chemotherapy | 1 | 0.82 | 1.3 ( 0.77 , 2.21 ) | 1.4 ( 0.77 , 2.21 ) | 0.93 ( 0.77 , 2.21 ) | 0.561 |
| PARP inhibitor + Platinum-based chemotherapy:Platinum-based chemotherapy | 2 | 1 | 1.16 ( 0.79 , 1.7 ) | 1.16 ( 0.79 , 1.7 ) | NA ( 0.79 , 1.7 ) | NA |
| **Decreased appetite** |  |  |  |  |  |  |
| Comparisons | Number of studies providing direct evidence | Direct evidence proportion | Network estimates | Direct estimates | Indirect estimates | Inconsistency P-value |
| PARP inhibitor:Non-Platinum-based chemotherapy | 3 | 1 | 1.42 ( 0.87 , 2.31 ) | 1.42 ( 0.87 , 2.31 ) | NA ( 0.87 , 2.31 ) | NA |
| PARP inhibitor + Platinum-based chemotherapy:Non-Platinum-based chemotherapy | 0 | 0 | 1.45 ( 0.59 , 3.57 ) | NA ( 0.59 , 3.57 ) | 1.45 ( 0.59 , 3.57 ) | NA |
| Platinum-based chemotherapy:Non-Platinum-based chemotherapy | 0 | 0 | 1.49 ( 0.6 , 3.68 ) | NA ( 0.6 , 3.68 ) | 1.49 ( 0.6 , 3.68 ) | NA |
| PARP inhibitor:PARP inhibitor + Platinum-based chemotherapy | 1 | 0.86 | 0.98 ( 0.46 , 2.09 ) | 0.91 ( 0.46 , 2.09 ) | 1.56 ( 0.46 , 2.09 ) | 0.633 |
| PARP inhibitor:Platinum-based chemotherapy | 1 | 0.85 | 0.95 ( 0.44 , 2.05 ) | 1.03 ( 0.44 , 2.05 ) | 0.61 ( 0.44 , 2.05 ) | 0.633 |
| PARP inhibitor + Platinum-based chemotherapy:Platinum-based chemotherapy | 2 | 1 | 0.98 ( 0.57 , 1.66 ) | 0.98 ( 0.57 , 1.66 ) | NA ( 0.57 , 1.66 ) | NA |
| **Fatigue** |  |  |  |  |  |  |
| Comparisons | Number of studies providing direct evidence | Direct evidence proportion | Network estimates | Direct estimates | Indirect estimates | Inconsistency P-value |
| PARP inhibitor:Non-Platinum-based chemotherapy | 3 | 1 | 1.13 ( 0.96 , 1.34 ) | 1.13 ( 0.96 , 1.34 ) | NA ( 0.96 , 1.34 ) | NA |
| PARP inhibitor + Platinum-based chemotherapy:Non-Platinum-based chemotherapy | 0 | 0 | 1.29 ( 0.94 , 1.76 ) | NA ( 0.94 , 1.76 ) | 1.29 ( 0.94 , 1.76 ) | NA |
| Platinum-based chemotherapy:Non-Platinum-based chemotherapy | 0 | 0 | 1.36 ( 1 , 1.85 ) | NA ( 1 , 1.85 ) | 1.36 ( 1 , 1.85 ) | NA |
| PARP inhibitor:PARP inhibitor + Platinum-based chemotherapy | 1 | 0.81 | 0.88 ( 0.67 , 1.14 ) | 0.94 ( 0.67 , 1.14 ) | 0.66 ( 0.67 , 1.14 ) | 0.321 |
| PARP inhibitor:Platinum-based chemotherapy | 1 | 0.9 | 0.83 ( 0.64 , 1.08 ) | 0.8 ( 0.64 , 1.08 ) | 1.23 ( 0.64 , 1.08 ) | 0.321 |
| PARP inhibitor + Platinum-based chemotherapy:Platinum-based chemotherapy | 2 | 1 | 0.95 ( 0.82 , 1.1 ) | 0.95 ( 0.82 , 1.1 ) | NA ( 0.82 , 1.1 ) | NA |
| **Headache** |  |  |  |  |  |  |
| Comparisons | Number of studies providing direct evidence | Direct evidence proportion | Network estimates | Direct estimates | Indirect estimates | Inconsistency P-value |
| PARP inhibitor:Non-Platinum-based chemotherapy | 3 | 1 | 1.52 ( 1.16 , 2.01 ) | 1.52 ( 1.16 , 2.01 ) | NA ( 1.16 , 2.01 ) | NA |
| PARP inhibitor + Platinum-based chemotherapy:Non-Platinum-based chemotherapy | 0 | 0 | 1.82 ( 1.13 , 2.92 ) | NA ( 1.13 , 2.92 ) | 1.82 ( 1.13 , 2.92 ) | NA |
| Platinum-based chemotherapy:Non-Platinum-based chemotherapy | 0 | 0 | 1.75 ( 1.08 , 2.82 ) | NA ( 1.08 , 2.82 ) | 1.75 ( 1.08 , 2.82 ) | NA |
| PARP inhibitor:PARP inhibitor + Platinum-based chemotherapy | 1 | 0.86 | 0.84 ( 0.57 , 1.24 ) | 0.82 ( 0.57 , 1.24 ) | 0.98 ( 0.57 , 1.24 ) | 0.75 |
| PARP inhibitor:Platinum-based chemotherapy | 1 | 0.83 | 0.87 ( 0.59 , 1.29 ) | 0.9 ( 0.59 , 1.29 ) | 0.76 ( 0.59 , 1.29 ) | 0.75 |
| PARP inhibitor + Platinum-based chemotherapy:Platinum-based chemotherapy | 2 | 1 | 1.04 ( 0.84 , 1.28 ) | 1.04 ( 0.84 , 1.28 ) | NA ( 0.84 , 1.28 ) | NA |
| **Back pain** |  |  |  |  |  |  |
| Comparisons | Number of studies providing direct evidence | Direct evidence proportion | Network estimates | Direct estimates | Indirect estimates | Inconsistency P-value |
| PARP inhibitor:Non-Platinum-based chemotherapy | 3 | 1 | 1.48 ( 1.05 , 2.08 ) | 1.48 ( 1.05 , 2.08 ) | NA ( 1.05 , 2.08 ) | NA |
| PARP inhibitor + Platinum-based chemotherapy:Non-Platinum-based chemotherapy | 0 | 0 | 1.52 ( 0.87 , 2.64 ) | NA ( 0.87 , 2.64 ) | 1.52 ( 0.87 , 2.64 ) | NA |
| Platinum-based chemotherapy:Non-Platinum-based chemotherapy | 0 | 0 | 1.56 ( 0.89 , 2.76 ) | NA ( 0.89 , 2.76 ) | 1.56 ( 0.89 , 2.76 ) | NA |
| PARP inhibitor:PARP inhibitor + Platinum-based chemotherapy | 1 | 0.88 | 0.97 ( 0.63 , 1.5 ) | 0.86 ( 0.63 , 1.5 ) | 2.42 ( 0.63 , 1.5 ) | 0.126 |
| PARP inhibitor:Platinum-based chemotherapy | 1 | 0.8 | 0.94 ( 0.6 , 1.48 ) | 1.13 ( 0.6 , 1.48 ) | 0.47 ( 0.6 , 1.48 ) | 0.126 |
| PARP inhibitor + Platinum-based chemotherapy:Platinum-based chemotherapy | 2 | 1 | 0.97 ( 0.73 , 1.29 ) | 0.97 ( 0.73 , 1.29 ) | NA ( 0.73 , 1.29 ) | NA |
| **Alopecia** |  |  |  |  |  |  |
| PARP inhibitor:Non-platinum-based chemotherapy | 2 | 1 | 0.67 ( 0.42 , 1.09 ) | 0.67 ( 0.42 , 1.09 ) | NA ( 0.42 , 1.09 ) | NA |
| PARP inhibitor + Platinum-based chemotherapy:Non-platinum-based chemotherapy | 0 | 0 | 4.07 ( 1.71 , 9.68 ) | NA ( 1.71 , 9.68 ) | 4.07 ( 1.71 , 9.68 ) | NA |
| Platinum-based chemotherapy:Non-platinum-based chemotherapy | 0 | 0 | 3.68 ( 1.54 , 8.76 ) | NA ( 1.54 , 8.76 ) | 3.68 ( 1.54 , 8.76 ) | NA |
| PARP inhibitor:PARP inhibitor + Platinum-based chemotherapy | 1 | 0.95 | 0.17 ( 0.08 , 0.34 ) | 0.16 ( 0.08 , 0.34 ) | 0.26 ( 0.08 , 0.34 ) | 0.782 |
| PARP inhibitor:Platinum-based chemotherapy | 1 | 0.94 | 0.18 ( 0.09 , 0.38 ) | 0.19 ( 0.09 , 0.38 ) | 0.12 ( 0.09 , 0.38 ) | 0.782 |
| PARP inhibitor + Platinum-based chemotherapy:Platinum-based chemotherapy | 2 | 1 | 1.11 ( 0.79 , 1.55 ) | 1.11 ( 0.79 , 1.55 ) | NA ( 0.79 , 1.55 ) | NA |

**Appendix 7: Comparison-adjusted funnel plots**

**
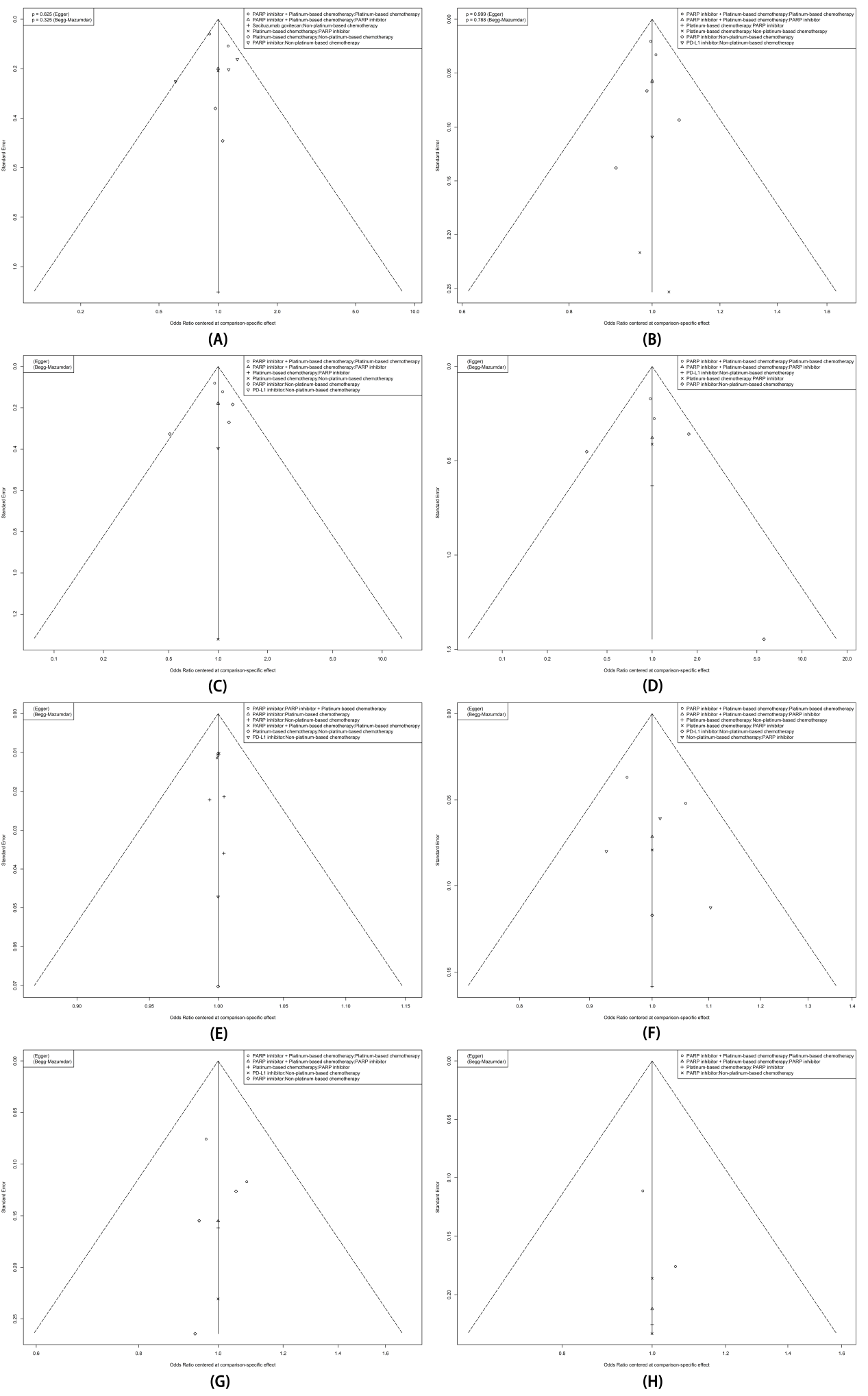
**

Comparison-adjusted funnel plots for efficacy outcomes: (A) ORR; (B) 3-month PFS; (C) 12-month PFS; (D) 24-month PFS; (E) 3-month OS; (F) 12-month OS; (G) 24-month OS (H) 36-month OS


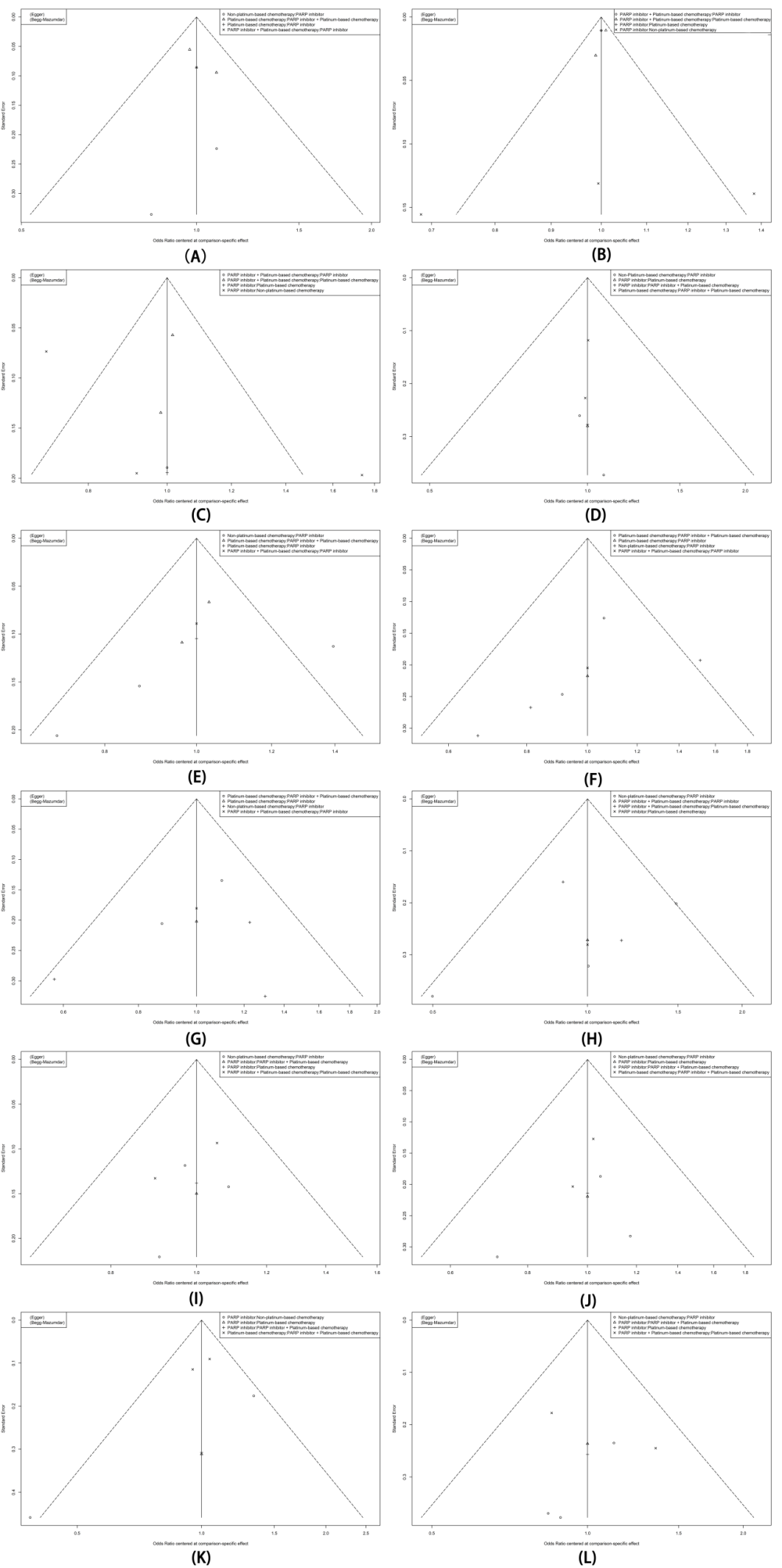


Comparison-adjusted funnel plots for safety outcomes: (A) Thrombocytopenia; (B)Neutropenia; (C) Aganemia; (D) Leukopenia; (E) Nausea; (F) Vomiting; (G) Constipation; (H)Decreased appetite; (I)Fatigue; (J) Headache; (K) Alopecia; (L) Back pain

**Appendix 8: Certainty of evidence for direct, indirect and network estimates, The GRADE assessments**

| Comparisons | Direct estimate | Certainty | Indirect estimate | Certainty | Network estimate | Certainty |
| --- | --- | --- | --- | --- | --- | --- |
| **Outcome: ORR** |  |  |  |  |  |  |
| PARP inhibitor:Non-platinum-based chemotherapy | 1.84 ( 0.28, 0.94 ) | Moderate | 0.92 ( -0.93, 0.77 ) | Moderate | 1.68 ( 0.21, 0.83 ) | Moderate |
| Platinum-based chemotherapy:Non-platinum-based chemotherapy | 2.11 ( 0.1, 1.39 ) | Low | 4.2 ( 0.79, 2.07 ) | Moderate | 2.98 ( 0.64, 1.55 ) | Moderate |
| Sacituzumab govitecan:Non-platinum-based chemotherapy | 3.38 ( -0.98, 3.42 ) | Moderate | / | / | 3.38 ( -0.98, 3.42 ) | Low |
| ARP inhibitor + Platinum-based chemotherapy:Non-platinum-based chemotherapy | / | / | 3.52 ( 0.76, 1.76 ) | Moderate | 3.52 ( 0.76, 1.76 ) | Moderate |
| PARP inhibitor:Platinum-based chemotherapy | 0.47 ( -1.34, -0.18 ) | High | 0.73 ( -0.99, 0.35 ) | Moderate | 0.56 ( -1.01, -0.14 ) | High |
| PARP inhibitor:Sacituzumab govitecan | / | / | 0.5 ( -2.92, 1.52 ) | Moderate | 0.5 ( -2.92, 1.52 ) | Low |
| Platinum-based chemotherapy:Sacituzumab govitecan | / | / | 0.88 ( -2.37, 2.12 ) | Moderate | 0.88 ( -2.37, 2.12 ) | Low |
| PARP inhibitor + Platinum-based chemotherapy:Platinum-based chemotherapy | 1.13 ( -0.19, 0.43 ) | High | 4.09 ( -0.26, 3.07 ) | High | 1.18 ( -0.14, 0.47 ) | High |
| PARP inhibitor:PARP inhibitor + Platinum-based chemotherapy | 0.37 ( -1.57, -0.44 ) | High | 0.85 ( -1, 0.67 ) | High | 0.48 ( -1.21, -0.27 ) | High |
| PARP inhibitor + Platinum-based chemotherapy:Sacituzumab govitecan | / | / | 1.04 ( -2.21, 2.3 ) | Moderate | 1.04 ( -2.21, 2.3 ) | Low |
| **Outcome: PFS-3 month** |  |  |  |  |  |  |
| PD-L1 inhibitor:Non-platinum-based chemotherapy | 1.15 ( -0.07, 0.36 ) | Low | / | / | 1.15 ( -0.07, 0.36 ) | Low |
| PARP inhibitor:Non-platinum-based chemotherapy | 1.28 ( 0.15, 0.35 ) | Moderate | 1.24 ( -0.12, 0.56 ) | Low | 1.28 ( 0.15, 0.34 ) | Moderate |
| Platinum-based chemotherapy:Non-platinum-based chemotherapy | 1.48 ( 0.07, 0.71 ) | Low | 1.53 ( 0.27, 0.57 ) | Moderate | 1.52 ( 0.28, 0.55 ) | Moderate |
| PARP inhibitor + Platinum-based chemotherapy:Non-platinum-based chemotherapy | / | / | 1.53 ( 0.29, 0.56 ) | Moderate | 1.53 ( 0.29, 0.56 ) | Moderate |
| PARP inhibitor:PD-L1 inhibitor | / | / | 1.11 ( -0.13, 0.34 ) | Low | 1.11 ( -0.13, 0.34 ) | Low |
| PD-L1 inhibitor:Platinum-based chemotherapy | / | / | 0.76 ( -0.53, -0.02 ) | Low | 0.76 ( -0.53, -0.02 ) | Low |
| PARP inhibitor + Platinum-based chemotherapy:PD-L1 inhibitor | / | / | 1.33 ( 0.03, 0.54 ) | Low | 1.33 ( 0.03, 0.54 ) | Low |
| PARP inhibitor:Platinum-based chemotherapy | 0.85 ( -0.28, -0.05 ) | High | 0.83 ( -0.44, 0.07 ) | Low | 0.84 ( -0.27, -0.06 ) | High |
| PARP inhibitor:PARP inhibitor + Platinum-based chemotherapy | 0.83 ( -0.3, -0.08 ) | High | 0.88 ( -0.43, 0.17 ) | High | 0.83 ( -0.29, -0.08 ) | High |
| PARP inhibitor + Platinum-based chemotherapy:Platinum-based chemotherapy | 1.01 ( -0.02, 0.05 ) | High | 1.07 ( -0.52, 0.65 ) | High | 1.01 ( -0.02, 0.05 ) | High |
| **Outcome: PFS-12 month** |  |  |  |  |  |  |
| PD-L1 inhibitor:Non-platinum-based chemotherapy | 1.37 ( -0.51, 1.15 ) | Low | / | / | 1.37 ( -0.51, 1.15 ) | Low |
| PARP inhibitor:Non-platinum-based chemotherapy | 1.53 ( 0.1, 0.75 ) | Moderate | 0.72 ( -2.97, 2.32 ) | Low | 1.51 ( 0.09, 0.73 ) | Moderate |
| Platinum-based chemotherapy:Non-platinum-based chemotherapy | 1.32 ( -2.33, 2.89 ) | Low | 2.79 ( 0.48, 1.57 ) | Moderate | 2.7 ( 0.46, 1.53 ) | Moderate |
| PARP inhibitor + Platinum-based chemotherapy:Non-platinum-based chemotherapy | / | / | 3.05 ( 0.58, 1.65 ) | Moderate | 3.05 ( 0.58, 1.65 ) | Moderate |
| PARP inhibitor:PD-L1 inhibitor | / | / | 1.1 ( -0.79, 0.98 ) | Low | 1.1 ( -0.79, 0.98 ) | Low |
| PD-L1 inhibitor:Platinum-based chemotherapy | / | / | 0.51 ( -1.66, 0.31 ) | Low | 0.51 ( -1.66, 0.31 ) | Low |
| PARP inhibitor + Platinum-based chemotherapy:PD-L1 inhibitor | / | / | 2.22 ( -0.19, 1.78 ) | Low | 2.22 ( -0.19, 1.78 ) | Low |
| PARP inhibitor:Platinum-based chemotherapy | 0.57 ( -1.03, -0.1 ) | High | 0.51 ( -1.87, 0.52 ) | Low | 0.56 ( -1.02, -0.15 ) | High |
| PARP inhibitor:PARP inhibitor + Platinum-based chemotherapy | 0.47 ( -1.2, -0.3 ) | High | 0.75 ( -1.65, 1.07 ) | High | 0.5 ( -1.13, -0.27 ) | High |
| PARP inhibitor + Platinum-based chemotherapy:Platinum-based chemotherapy | 1.12 ( -0.13, 0.36 ) | High | 4.47 ( -3.43, 6.42 ) | High | 1.13 ( -0.13, 0.37 ) | High |
| **Outcome: PFS-24 month** |  |  |  |  |  |  |
| PD-L1 inhibitor:Non-platinum-based chemotherapy | 3.81 ( -0.22, 2.89 ) | Low | / | / | 3.81 ( -0.22, 2.89 ) | Very low |
| PARP inhibitor:Non-platinum-based chemotherapy | 1.69 ( -0.31, 1.36 ) | Moderate | / | / | 1.69 ( -0.31, 1.36 ) | Moderate |
| Platinum-based chemotherapy:Non-platinum-based chemotherapy | / | / | 3.32 ( -0.23, 2.63 ) | Low | 3.32 ( -0.23, 2.63 ) | Very low |
| PARP inhibitor + Platinum-based chemotherapy:Non-platinum-based chemotherapy | / | / | 5.8 ( 0.35, 3.17 ) | Moderate | 5.8 ( 0.35, 3.17 ) | Low |
| PARP inhibitor:PD-L1 inhibitor | / | / | 0.44 ( -2.58, 0.95 ) | Low | 0.44 ( -2.58, 0.95 ) | Very low |
| PD-L1 inhibitor:Platinum-based chemotherapy | / | / | 1.15 ( -1.97, 2.25 ) | Low | 1.15 ( -1.97, 2.25 ) | Low |
| PARP inhibitor + Platinum-based chemotherapy:PD-L1 inhibitor | / | / | 1.52 ( -1.68, 2.52 ) | Low | 1.52 ( -1.68, 2.52 ) | Very low |
| PARP inhibitor:Platinum-based chemotherapy | 0.52 ( -1.9, 0.58 ) | High | 0.45 ( -4.11, 2.5 ) | High | 0.51 ( -1.84, 0.48 ) | High |
| PARP inhibitor:PARP inhibitor + Platinum-based chemotherapy | 0.29 ( -2.44, -0.06 ) | High | 0.34 ( -4.83, 2.65 ) | High | 0.29 ( -2.37, -0.1 ) | Moderate |
| PARP inhibitor + Platinum-based chemotherapy:Platinum-based chemotherapy | 1.75 ( -0.18, 1.29 ) | High | / | / | 1.75 ( -0.18, 1.29 ) | High |
| **Outcome: OS-3 month** |  |  |  |  |  |  |
| PD-L1 inhibitor:Platinum-based chemotherapy | / | / | 0.96 ( -0.14, 0.06 ) | Low | 0.96 ( -0.14, 0.06 ) | Low |
| PARP inhibitor + Platinum-based chemotherapy:Platinum-based chemotherapy | 1.01 ( -0.01, 0.02 ) | High | 1.04 ( -0.28, 0.35 ) | High | 1.01 ( -0.01, 0.02 ) | High |
| Platinum-based chemotherapy:Non-platinum-based chemotherapy | 1.02 ( -0.12, 0.15 ) | Low | 1.03 ( 0, 0.06 ) | Moderate | 1.03 ( 0, 0.06 ) | Moderate |
| PARP inhibitor:Platinum-based chemotherapy | 1.01 ( -0.01, 0.03 ) | High | 1.01 ( -0.05, 0.07 ) | Low | 1.01 ( -0.01, 0.03 ) | High |
| PARP inhibitor + Platinum-based chemotherapy:PD-L1 inhibitor | / | / | 1.05 ( -0.05, 0.15 ) | Low | 1.05 ( -0.05, 0.15 ) | Low |
| PD-L1 inhibitor:Non-platinum-based chemotherapy | 0.99 ( -0.1, 0.08 ) | Low | / | / | 0.99 ( -0.1, 0.08 ) | Low |
| PARP inhibitor:PD-L1 inhibitor | / | / | 1.05 ( -0.05, 0.15 ) | Low | 1.05 ( -0.05, 0.15 ) | Low |
| PARP inhibitor + Platinum-based chemotherapy:Non-platinum-based chemotherapy | / | / | 1.04 ( 0, 0.07 ) | Moderate | 1.04 ( 0, 0.07 ) | Moderate |
| PARP inhibitor:PARP inhibitor + Platinum-based chemotherapy | 1 ( -0.02, 0.02 ) | High | 1 ( -0.05, 0.06 ) | High | 1 ( -0.02, 0.02 ) | High |
| PARP inhibitor:Non-platinum-based chemotherapy | 1.04 ( 0.01, 0.07 ) | Moderate | 1.03 ( -0.11, 0.17 ) | Low | 1.04 ( 0.01, 0.07 ) | Moderate |
| **Outcome: OS-12 month** |  |  |  |  |  |  |
| PD-L1 inhibitor:Platinum-based chemotherapy | / | / | 0.97 ( -0.34, 0.27 ) | Low | 0.97 ( -0.34, 0.27 ) | Low |
| PARP inhibitor + Platinum-based chemotherapy:Platinum-based chemotherapy | 1.05 ( -0.05, 0.14 ) | High | 1.58 ( -0.17, 1.08 ) | High | 1.06 ( -0.04, 0.15 ) | High |
| Platinum-based chemotherapy:Non-platinum-based chemotherapy | 0.92 ( -0.41, 0.24 ) | Low | 1.18 ( -0.04, 0.37 ) | Moderate | 1.1 ( -0.08, 0.27 ) | Moderate |
| PARP inhibitor:Platinum-based chemotherapy | 0.85 ( -0.34, 0.03 ) | High | 0.9 ( -0.39, 0.17 ) | Low | 0.87 ( -0.3, 0.01 ) | High |
| PARP inhibitor + Platinum-based chemotherapy:PD-L1 inhibitor | / | / | 1.09 ( -0.22, 0.39 ) | Low | 1.09 ( -0.22, 0.39 ) | Low |
| PD-L1 inhibitor:Non-platinum-based chemotherapy | 1.07 ( -0.19, 0.31 ) | Low | / | / | 1.07 ( -0.19, 0.31 ) | Low |
| PARP inhibitor:PD-L1 inhibitor | / | / | 0.9 ( -0.38, 0.16 ) | Low | 0.9 ( -0.38, 0.16 ) | Low |
| PARP inhibitor + Platinum-based chemotherapy:Non-platinum-based chemotherapy | / | / | 1.16 ( -0.02, 0.33 ) | Moderate | 1.16 ( -0.02, 0.33 ) | Moderate |
| PARP inhibitor:PARP inhibitor + Platinum-based chemotherapy | 0.77 ( -0.43, -0.09 ) | High | 1.06 ( -0.29, 0.41 ) | High | 0.82 ( -0.35, -0.04 ) | High |
| PARP inhibitor:Non-platinum-based chemotherapy | 0.97 ( -0.13, 0.08 ) | Moderate | 0.76 ( -0.64, 0.09 ) | Low | 0.96 ( -0.15, 0.06 ) | Moderate |
| **Outcome: OS-24 month** |  |  |  |  |  |  |
| PD-L1 inhibitor:Platinum-based chemotherapy | / | / | 0.91 ( -0.66, 0.48 ) | Low | 0.91 ( -0.66, 0.48 ) | Low |
| PARP inhibitor + Platinum-based chemotherapy:Platinum-based chemotherapy | 1.06 ( -0.07, 0.18 ) | High | / | / | 1.06 ( -0.07, 0.18 ) | High |
| Platinum-based chemotherapy:Non-platinum-based chemotherapy | / | / | 1.66 ( 0.16, 0.86 ) | Moderate | 1.66 ( 0.16, 0.86 ) | Moderate |
| PARP inhibitor:Platinum-based chemotherapy | 0.67 ( -0.72, -0.09 ) | High | 0.46 ( -1.63, 0.06 ) | High | 0.64 ( -0.75, -0.15 ) | High |
| PARP inhibitor + Platinum-based chemotherapy:PD-L1 inhibitor | / | / | 1.16 ( -0.42, 0.72 ) | Low | 1.16 ( -0.42, 0.72 ) | Low |
| PD-L1 inhibitor:Non-platinum-based chemotherapy | 1.52 ( -0.03, 0.87 ) | Low | / | / | 1.52 ( -0.03, 0.87 ) | Low |
| PARP inhibitor:PD-L1 inhibitor | / | / | 0.7 ( -0.85, 0.13 ) | Low | 0.7 ( -0.85, 0.13 ) | Low |
| PARP inhibitor + Platinum-based chemotherapy:Non-platinum-based chemotherapy | / | / | 1.76 ( 0.22, 0.91 ) | Moderate | 1.76 ( 0.22, 0.91 ) | Moderate |
| PARP inhibitor:PARP inhibitor + Platinum-based chemotherapy | 0.58 ( -0.85, -0.24 ) | High | 0.94 ( -1.17, 1.03 ) | High | 0.6 ( -0.8, -0.22 ) | High |
| PARP inhibitor:Non-platinum-based chemotherapy | 1.06 ( -0.12, 0.24 ) | Moderate | / | / | 1.06 ( -0.12, 0.24 ) | Moderate |
| **Outcome: OS-36 month** |  |  |  |  |  |  |
| PARP inhibitor:Non-platinum-based chemotherapy | 1.31 ( -0.02, 0.55 ) | High | / | / | 1.31 ( -0.02, 0.55 ) | High |
| Platinum-based chemotherapy:Non-platinum-based chemotherapy | / | / | 1.91 ( 0.15, 1.14 ) | High | 1.91 ( 0.15, 1.14 ) | High |
| PARP inhibitor + Platinum-based chemotherapy:Non-platinum-based chemotherapy | / | / | 2.31 ( 0.35, 1.33 ) | High | 2.31 ( 0.35, 1.33 ) | High |
| PARP inhibitor:Platinum-based chemotherapy | 0.71 ( -0.79, 0.1 ) | High | 0.57 ( -1.6, 0.47 ) | High | 0.69 ( -0.78, 0.03 ) | High |
| PARP inhibitor:PARP inhibitor + Platinum-based chemotherapy | 0.55 ( -1.01, -0.18 ) | High | 0.74 ( -1.71, 1.11 ) | High | 0.57 ( -0.97, -0.17 ) | High |
| PARP inhibitor + Platinum-based chemotherapy:Platinum-based chemotherapy | 1.21 ( 0.01, 0.38 ) | High | / | / | 1.21 ( 0.01, 0.38 ) | High |

**Appendix 9: Results of sensitivity analysis**

Table 1 Rank the treatments by the surface under the cumulative ranking curve(SUCRA) values of efficacy outcomes

| Treatments | ORR | 3-month PFS | 12-month PFS |  | 3-month OS | 12-month OS | 24-month OS | 36-month OS |
| --- | --- | --- | --- | --- | --- | --- | --- | --- |
| PARP inhibitor + Platinum-based chemotherapy | 0.8195 | 0.8908 | 0.9072 | 0.8562 | 0.7735 | 0.8665 | 0.847 | 0.9477 |
| Platinum-based chemotherapy | 0.6755 | 0.8518 | 0.8062 | 0.6228 | 0.539 | 0.6818 | 0.7412 | 0.714 |
| Sacituzumab govitecan | 0.653 | NA | NA | NA | NA | NA | NA | NA |
| PARP inhibitor | 0.3182 | 0.4555 | 0.3938 | 0.2955 | 0.7852 | 0.1172 | 0.1875 | 0.3057 |
| Non-platinum-based chemotherapy | 0.0338 | 0.0312 | 0.06 | 0.0578 | 0.1568 | 0.3085 | 0.092 | 0.0327 |
| PD-L1 inhibitor | NA | 0.2708 | 0.3328 | 0.6678 | 0.2455 | 0.526 | 0.6322 | NA |

Table 2 League tables of network estimates of odds ratios for efficacy outcome analyses after excluding the trial with somatic BRCA mutations (IMpassion130)

1. 3-month PFS(lower triangle); 12-month PFS(upper triangle)

| PARP inhibitor + Platinum-based chemotherapy | 1.13 (0.88,1.44) | **2.02 (1.31,3.10)** | **3.05 (1.79,5.19)** |
| --- | --- | --- | --- |
| 1.01 (0.98,1.05) | Platinum-based chemotherapy | **1.79 (1.16,2.76)** | **2.70 (1.58,4.62)** |
| **1.20 (1.08,1.33)** | **1.18 (1.07,1.32)** | PARP inhibitor | 1.51 (1.09,2.08) |
| **1.53 (1.34,1.76)** | **1.52 (1.33,1.74)** | **1.28 (1.16,1.41)** | Non-platinum-based chemotherapy |

1. 24-month PFS(lower triangle); 3-month OS(upper triangle)

| PARP inhibitor + Platinum-based chemotherapy | 1.01 (0.99,1.02) | 1.00 (0.98,1.02) | 1.04 (1.00,1.07) |
| --- | --- | --- | --- |
| **1.75 (0.84, 3.63)** | Platinum-based chemotherapy | 0.99 (0.97,1.01) | 1.03 (1.00,1.06) |
| **3.44 (1.10,10.72)** | 1.97 (0.62, 6.27) | PARP inhibitor | **1.04 (1.01,1.07)** |
| **5.80 (1.42,23.77)** | 3.32 (0.80,13.84) | 1.69 (0.73, 3.88) | Non-platinum-based chemotherapy |

1. 12-month OS(lower triangle); 24 month OS(upper triangle)

| PARP inhibitor + Platinum-based chemotherapy | 1.06 (0.94,1.20) | **1.76 (1.25,2.49)** | **1.66 (1.24,2.23)** |
| --- | --- | --- | --- |
| 1.06 (0.96,1.16) | Platinum-based chemotherapy | **1.66 (1.18,2.36)** | **1.57 (1.17,2.11)** |
| 1.16 (0.98,1.39) | 1.10 (0.93,1.31) | Non-platinum-based chemotherapy | 0.94 (0.79,1.13) |
| **1.22 (1.04,1.42)** | 1.15 (0.99,1.35) | 1.05 (0.94,1.16) | PARP inhibitor |
